# Supplementary material for: Visualizing single-molecule conformational transition and binding dynamics of intrinsically disordered proteins
Source: Nat Commun. 2023 Aug 25;14:5203. doi: 10.1038/s41467-023-41018-x (PMC10457384; doi:10.1038/s41467-023-41018-x)
Supplement: Supplementary file 1 — Supplementary Information [file 41467_2023_41018_MOESM1_ESM.pdf]

Supplementary Information for

Visualizing single-molecule conformational transition and binding dynamics of intrinsically disordered proteins

Wenzhe Liu[1†], Limin Chen[2†], Dongbao Yin[1], Zhiheng Yang[1], Jianfei Feng[1], Qi Sun[1]*, Luhua Lai[1,2,3]*, and Xuefeng Guo[1,4,5]*

[1]Beijing National Laboratory for Molecular Sciences, College of Chemistry and Molecular Engineering, Peking University, 292 Chengfu Road, Haidian District, Beijing 100871, P. R. China.

[2]Peking-Tsinghua Center for Life Sciences, Peking University, Beijing 100871, P. R. China.

[3]Center for Quantitative Biology, Academy for Advanced Interdisciplinary Studies, Peking University, Beijing 100871, P. R. China.

[4]Center of Single-Molecule Sciences, Institute of Modern Optics, Frontiers Science Center for New Organic Matter, College of Electronic Information and Optical Engineering, Nankai University, 38 Tongyan Road, Jinnan District, Tianjin 300350, P. R. China.

[5]National Biomedical Imaging Center, Peking University, Beijing 100871, P. R. China.

[†]These authors contributed equally to this work.

*Corresponding authors. E-mail: guoxf@pku.edu.cn (Xuefeng Guo); lhlai@pku.edu.cn (Luhua Lai); qsun2015@pku.edu.cn (Qi Sun).

Table of Contents

Supplementary Note 1: Materials

1. MS spectrum of LC46 provided by the supplier.
2. HPLC spectrum of LC46 provided by the supplier.
3. MS spectrum of DQ47 provided by the supplier.
4. HPLC spectrum of DQ47 provided by the supplier.
5. MS spectrum of 10074-A4.
6. ^{1}H NMR of 10074-A4 in DMSO-d_6.
7. ^{13}C NMR of 10074-A4 in DMSO-d_6.
8. HPLC spectrum of 10074-A4.
9. MS spectrum of PKUMDL-YC-1205.
10. ^{1}H NMR of PKUMDL-YC-1205 in DMSO-d_6.
11. ^{13}C NMR of PKUMDL-YC-1205 in DMSO-d_6.
12. HPLC spectrum of PKUMDL-YC-1205.

Supplementary Note 2: Device fabrication and characterization

13. Fabrication flowchart of a single-peptide modified device.
14. Characterization of SiNW-FET device arrays.
15. Electrical characterization of the SiNW-FET.
16. Schematic of the point modification procedure after wet-etching.
17. Fluorescence characterization of single-Myc-modified devices.
18. AFM image of a single-LC46 modified SiNW-FET device.

Supplementary Note 3: Real-time current measurements and dynamic analysis

19. PBS concentration-dependent experiments.
20. Real-time measurements of control devices and experimental devices.
21. Dimension information of a LC46 modified device.

22. Sensing principle of a *p*-type SiNW-FET device with c-Myc modification.

23. Temperature-dependent experiments of the c-Myc IDR folding process.

24. The dwell-time distributions of different current states at different temperatures.

25. Max concentration-dependent experiments at pH = 7.4.

26. Control experiments using molecule-linkage devices without LC46 modification.

27. Max concentration-dependent experiments at pH = 6.5.

28. Variation in the dwell-time mean values $\langle t \rangle$ of DQ47-LC46 binding kinetics.

29. Temperature-dependent experiments of Myc-Max binding process at pH = 7.4.

30. Surface plasmon resonance assay of LC46 with DQ47.

31. Experiments measuring different devices in 1 μM DQ47 solution at 37 ºC.

32. Dwell time distributions of the Myc-Max binding process at different temperatures.

33. 10074-A4 concentration-dependent experiments under the physiological condition.

34. PKUMDL-YC-1205 concentration-dependent experiments under the physiological condition.

35. Max concentration-dependent experiments in competition with 100 μM 10074-A4.

36. Max concentration-dependent experiments in competition with 100 μM PKUMDL-YC-1205.

37. k_{obs} at different DQ47 concentrations and different pHs.

Supplementary Note 4: Supplementary Tables

Supplementary Note 5: Supplementary References

Supplementary Note 1: Materials

c-Myc$_{370–414}$ IDR sequence with terminal cysteine (LC46):

LKRSFFALRDQIPELENNEKAPKVVILKKATAYILSVQAEEQKLIC

The number of amino acids: 46.

Molecular weight: 5287.25 Da.

Theoretical pI: 8.94.

Max$_{37–83}$ sequence (DQ47):

DHIKDSFHSLRDSVPSLQGEKASRAQILDKA TEYIQYMRRKNHTHQQ

The number of amino acids: 47.

Molecular weight: 5565.19 Da.

Theoretical pI: 9.31.

Samples of LC46 and DQ47 were synthesized by GL Biochem. Ltd., Shanghai, China with purity of more than 95%, which was confirmed by the supplier, using HPLC and MS (Supplementary Figs. 1–4). LC46 and DQ47 both carry +2e at pH 7.4. Compound 10074-A4 (Catalogue number: STK834743) was purchased from Vitas-M Laboratory, Ltd, which is commercially available from TopScience Co. (Shanghai, China), with the purity of more than 90%. The company provided ^{1}H NMR. The 1× PBS buffer was ordered from M&C Gene Technology (Catalogue number: CC008), which contains 8g/L NaCl, 0.2 g/L KCl, 1.44 g/L Na_2HPO_4 and 0.24 g/L KH_2PO_4 at pH 7.4. Compound PKUMDL-YC-1205 used in the experiments was purchased from Shenzhen Biochemilogic Technology Co. Ltd. The company provided NMR and LC-MS. Here, we rechecked the purity of PKUMDL-YC-1205 and 10074-A4 by performing HPLC, MS, ^{1}H and ^{13}C NMR. Both compounds are over 90% pure, which were determined by an Agilent 1206 Infinity high performance liquid chromatography instrument with a SB-C18 column (4.6 mm × 150 mm, 5 μm) with methanol and water with 0.1% formic acid as the mobile phase. The flow rate was 1 mL/min and the peak was detected at 254 nm. ^{1}H NMR spectra were recorded on a Bruker 500 MHz spectrometer. ^{13}C NMR spectra were recorded on a Bruker 400 MHz spectrometer. The chemical shift values (δ) are reported

in ppm relative to tetramethylsilane as the internal standard. ^{1}H spectra were represented as follows: chemical shift, multiplicity (s = singlet, d = doublet, t = triplet, m = multiplet), coupling constant (*J* values) in Hz and integration. ^{13}C NMR spectra were represented as follows: chemical shift, multiplicity (d = doublet, m = multiplet), coupling constant (*J* values) in Hz. High resolution mass spectra were recorded on a Bruker Solarix XR FTMS mass spectrometer using ESI (electrospray ionization). The fully assigned spectra of NMR, MS and HPLC have been provided as below (Supplementary Figs. 5−12).

Compound 10074-A4: ^{1}H NMR (500 MHz, DMSO-d_6) δ 8.32 (t, *J* = 1.7 Hz, 2H), 7.67 (d, *J* = 8.8 Hz, 2H), 7.49 (dt, *J* = 8.8, 1.6 Hz, 2H), 5.28 (dd, *J* = 5.6, 1.7 Hz, 1H), 4.47 (dd, *J* = 15.1, 4.0 Hz, 1H), 4.35 (dd, *J* = 15.0, 8.0 Hz, 1H), 4.22–4.16 (m, 1H), 4.14 (s, 2H), 3.73 (ddd, *J* = 13.5, 8.8, 1.5 Hz, 1H), 3.54 (dd, *J* = 13.6, 3.9 Hz, 1H). ^{13}C NMR (101 MHz, DMSO-d_6) δ 172.44, 172.12, 139.49, 126.09, 123.56, 122.48, 120.25, 111.76, 66.04, 46.96, 45.41, 33.81. HRMS (ESI): calcd for $C_{18}H_{14}Cl_2N_2O_3S$ $(M+H)^+$: 409.0175, found: 409.0176; $(M+NH_4)^+$: 426.0440, found: 426.0441.

Compound PKUMDL-YC-1205: ^{1}H NMR (500 MHz, DMSO-d_6) δ 12.70 (s, 1H), 8.60 (s, 1H), 7.90 (d, *J* = 7.6 Hz, 2H), 7.72 (d, *J* = 7.5 Hz, 2H), 7.59 (d, *J* = 8.3 Hz, 1H), 7.42 (q, *J* = 7.1 Hz, 2H), 7.31 (dd, *J* = 14.5, 7.4 Hz, 2H), 7.27–7.14 (m, 15H), 4.36 (dd, J = 10.3, 6.9 Hz, 1H) 4.32 – 4.20 (m, 3H), 2.67 (d, *J* = 7.1 Hz, 2H). ^{13}C NMR (101 MHz, DMSO-d_6) δ 173.25, 168.77, 155.82, 144.71, 143.79, 140.73, 128.58, 127.86 – 126.88 (m), 126.37, 125.22 (d, *J* = 4.2 Hz), 120.14, 69.45, 65.75, 51.06, 46.66, 38.04. HRMS (ESI): calcd for $C_{38}H_{32}N_2O_5$ $(M+H)^+$: 597.2384, found: 597.2389.

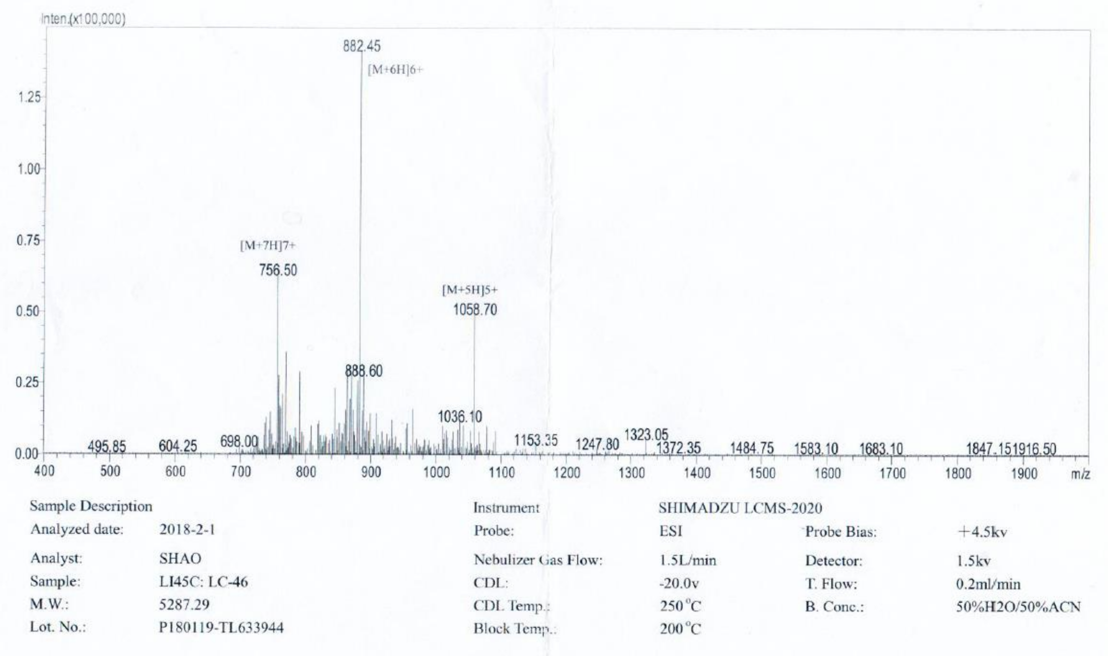

Supplementary Fig. 1 | MS spectrum of LC46 provided by the supplier.

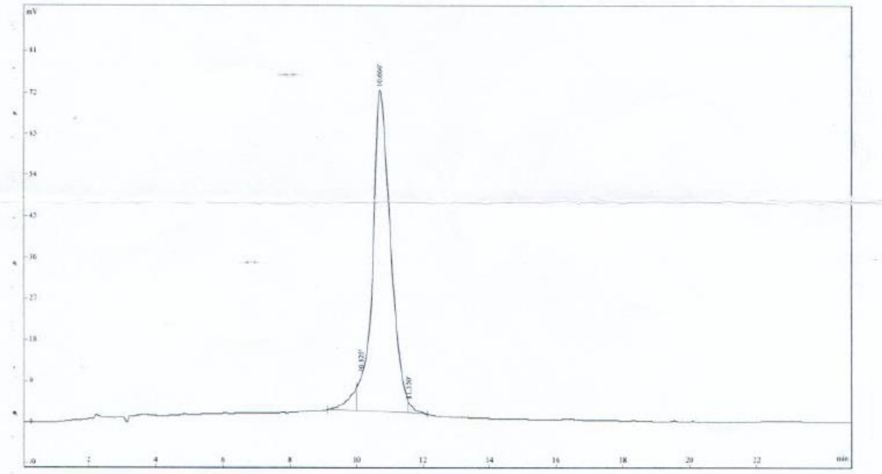

Rank	Time	Conc.	Area	Height
1	10.125	3.507	96411	7865
2	10.666	95.63	2628717	70447
3	11.550	0.8551	23504	2165
Total		100	2748632	80477

Supplementary Fig. 2 | HPLC spectrum of LC46 provided by the supplier.

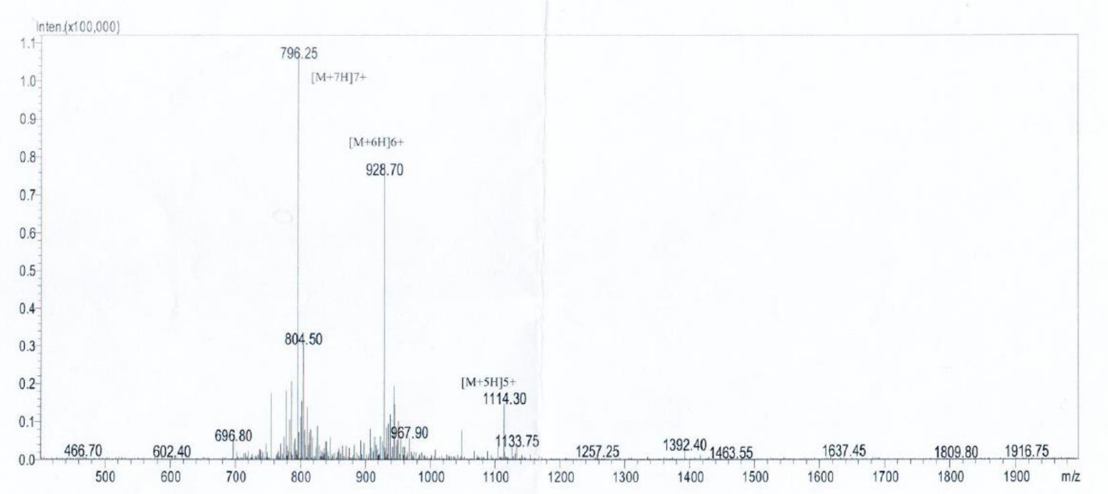

Sample Description		Instrument	SHIMADZU LCMS-2020		
Analyzed date:	2018-2-1	Probe:	ESI	Probe Bias:	+4.5kv
Analyst:	SHAO	Nebulizer Gas Flow:	1.5L/min	Detector:	1.5kv
Sample:	DQ47： DQ-47	CDL:	-20.0v	T. Flow:	0.2ml/min
M.W.:	5565.24	CDL Temp.:	250 °C	B. Conc.:	50%H2O/50%ACN
Lot. No.:	P180119-TL633945	Block Temp.:	200 °C		

Supplementary Fig. 3 | MS spectrum of DQ47 provided by the supplier.

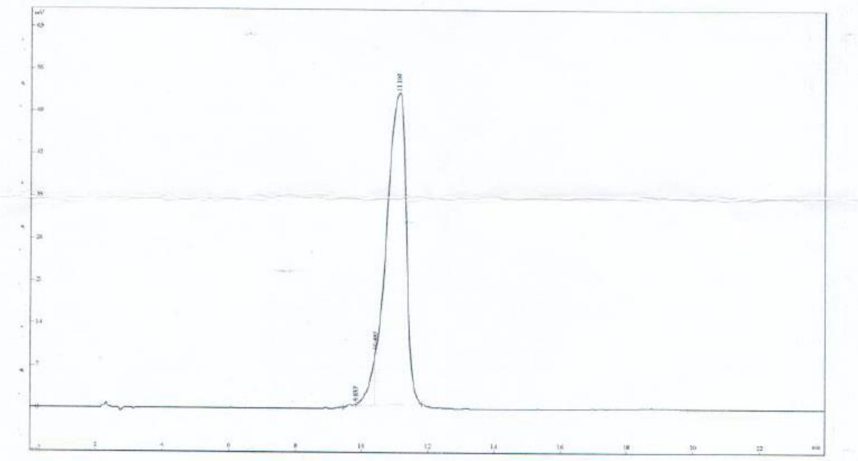

Rank	Time	Conc.	Area	Height
1	9.833	0.2492	5473	482
2	10.400	4.3404	95342	9214
3	11.104	95.4104	2095825	51570
Total		100	2196640	61266

Supplementary Fig. 4 | HPLC spectrum of DQ47 provided by the supplier.

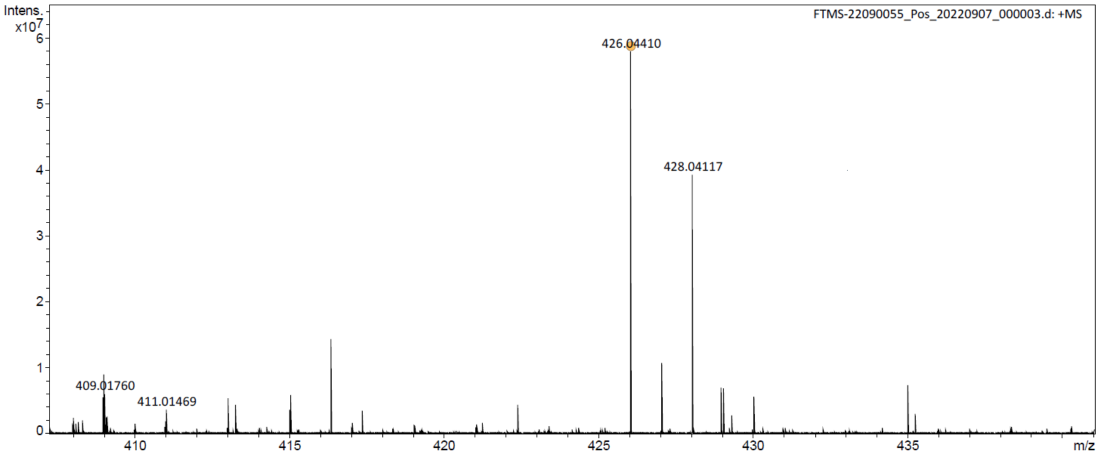

Meas. m/z	#	Ion Formula	Score	m/z	err [ppm]	Mean err [ppm]	mSigma	rdb	e⁻ Conf	N-Rule
409.017599	1	C18H15Cl2N2O3S	100.00	409.017495	-0.3	-0.7	35.4	20.0	even	ok
426.044102	1	C18H18Cl2N3O3S	100.00	426.044044	-0.1	-0.4	17.2	19.0	even	ok

Supplementary Fig. 5 | MS spectrum of 10074-A4.

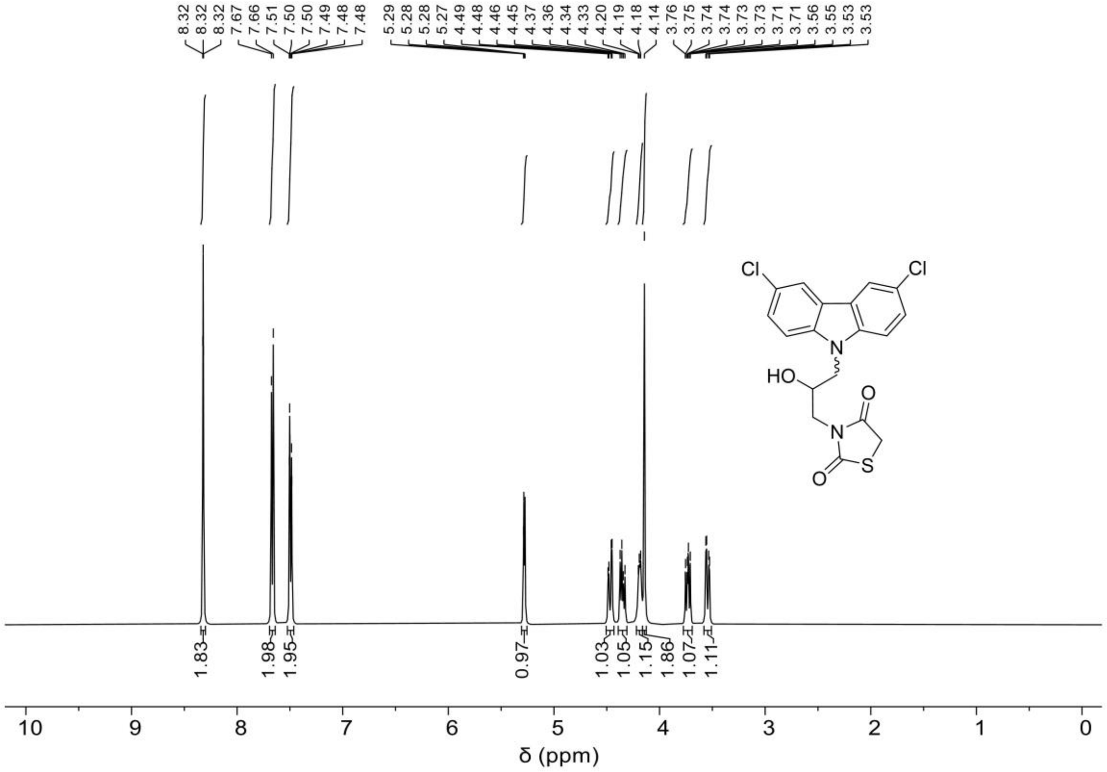

Supplementary Fig. 6 | ^{1}H NMR of 10074-A4 in DMSO-d_6.

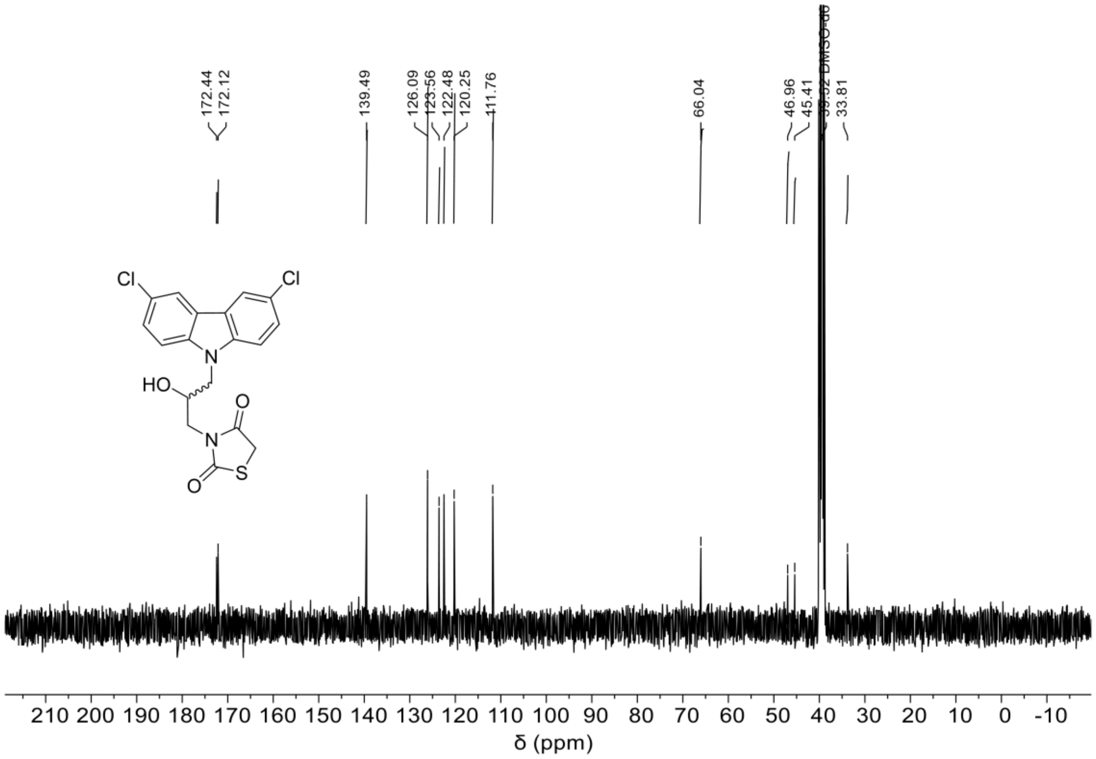

Supplementary Fig. 7 | ^{13}C NMR of 10074-A4 in DMSO-d_6.

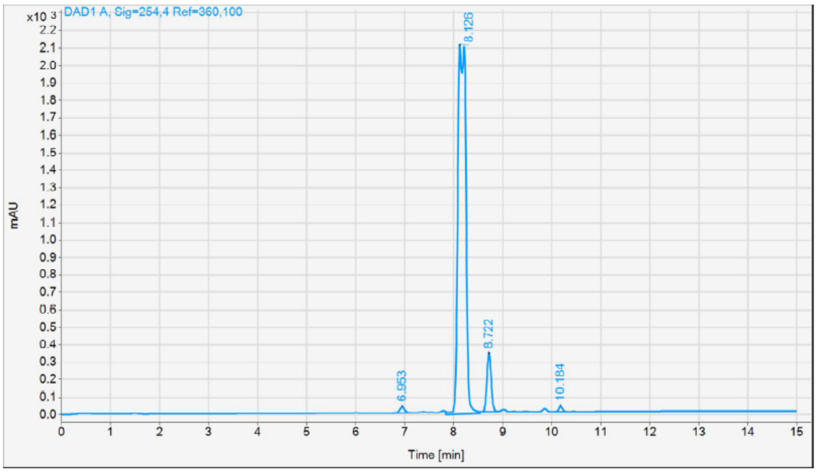

Signal: DAD1 A, Sig=254,4 Ref=360,100

RT [min]	Type	Width [min]	Area	Height	Area%	Name
6.953	BV	0.1112	229.3759	32.1635	0.8666	
8.126	MM	0.1899	24050.2598	2110.7236	90.8604	
8.722	BV	0.0958	2026.5587	329.1034	7.6562	
10.184	VV	0.0900	163.2565	27.9897	0.6168	
		Sum	26469.4509			

Supplementary Fig. 8 | HPLC spectrum of 10074-A4.

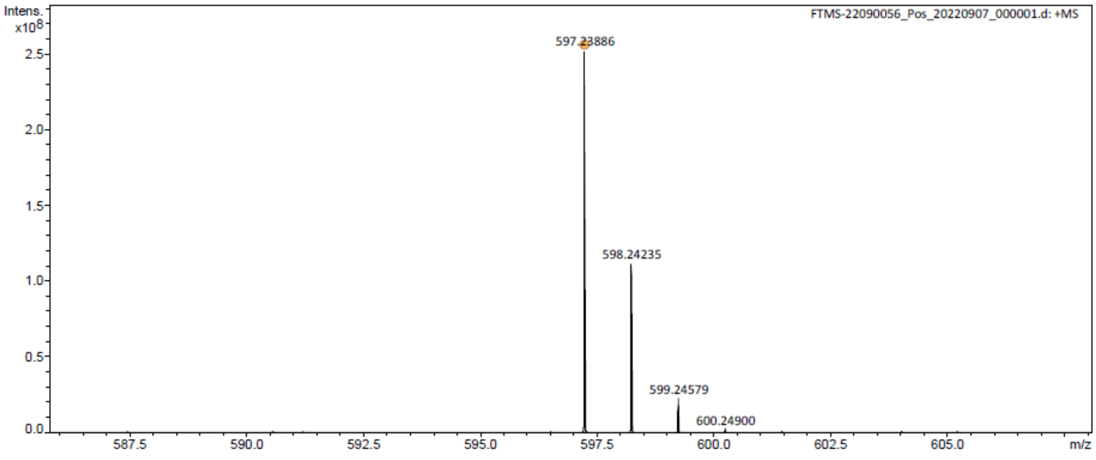

Meas. m/z	#	Ion Formula	Score	m/z	err [ppm]	Mean err [ppm]	mSigma	rdb	e⁻ Conf	N-Rule
597.238862	1	C38H33N2O5	100.00	597.238399	-0.8	-1.3	10.7	24.0	even	ok

Supplementary Fig. 9 | MS spectrum of PKUMDL-YC-1205.

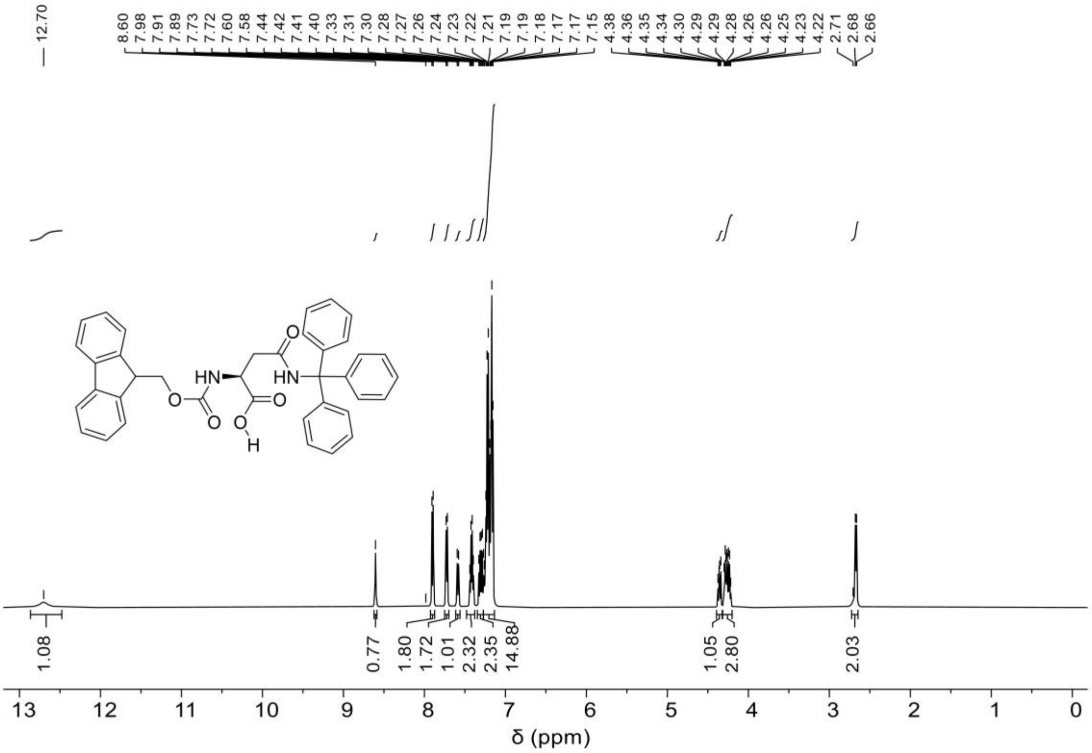

Supplementary Fig. 10 | ^{1}H NMR of PKUMDL-YC-1205 in DMSO-d_6.

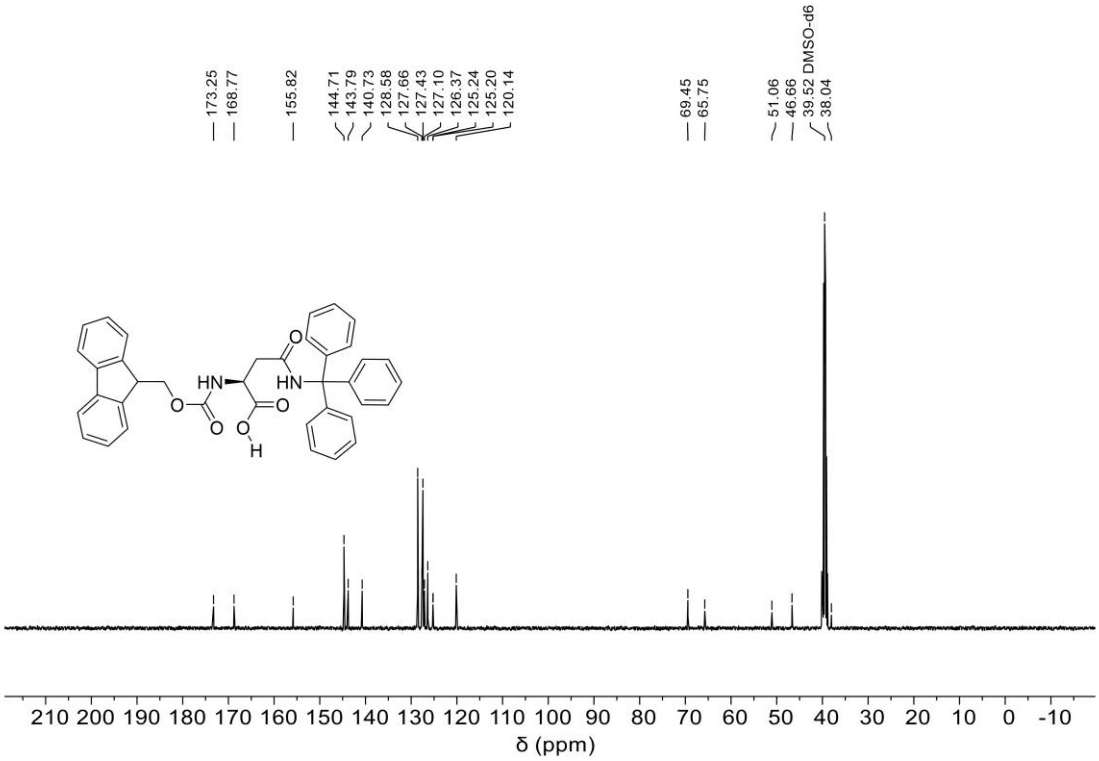

Supplementary Fig. 11 | ^{13}C NMR of 1205 in DMSO-*d*$_6$.

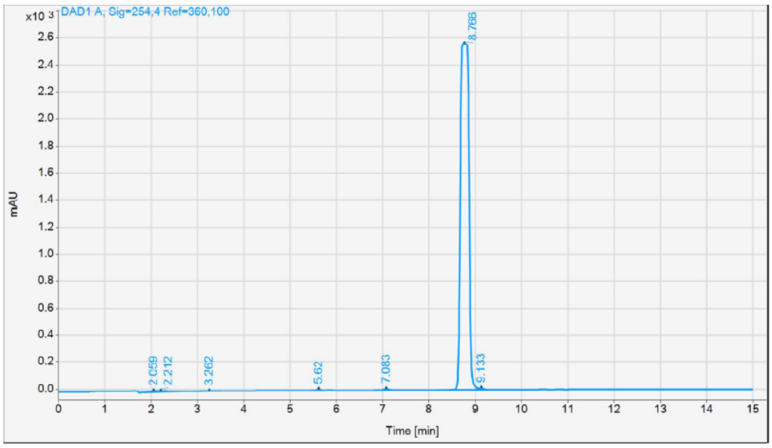

Signal: DAD1 A, Sig=254,4 Ref=360,100

RT [min]	Type	Width [min]	Area	Height	Area%	Name
2.059	BV	0.1283	77.9492	9.0674	0.2370	
2.212	VB	0.2151	151.0297	8.7618	0.4593	
3.262	BB	0.1654	28.1552	2.4617	0.0856	
5.620	BV	0.0911	50.6491	8.8125	0.1540	
7.083	BB	0.1209	88.3691	10.8587	0.2687	
8.766	BV	0.1858	32405.7129	2557.4395	98.5453	
9.133	VV	0.0995	82.2197	12.0686	0.2500	
		Sum	32884.0848			

Supplementary Fig. 12 | HPLC spectrum of PKUMDL-YC-1205.

Supplementary Note 2: Device fabrication and characterization

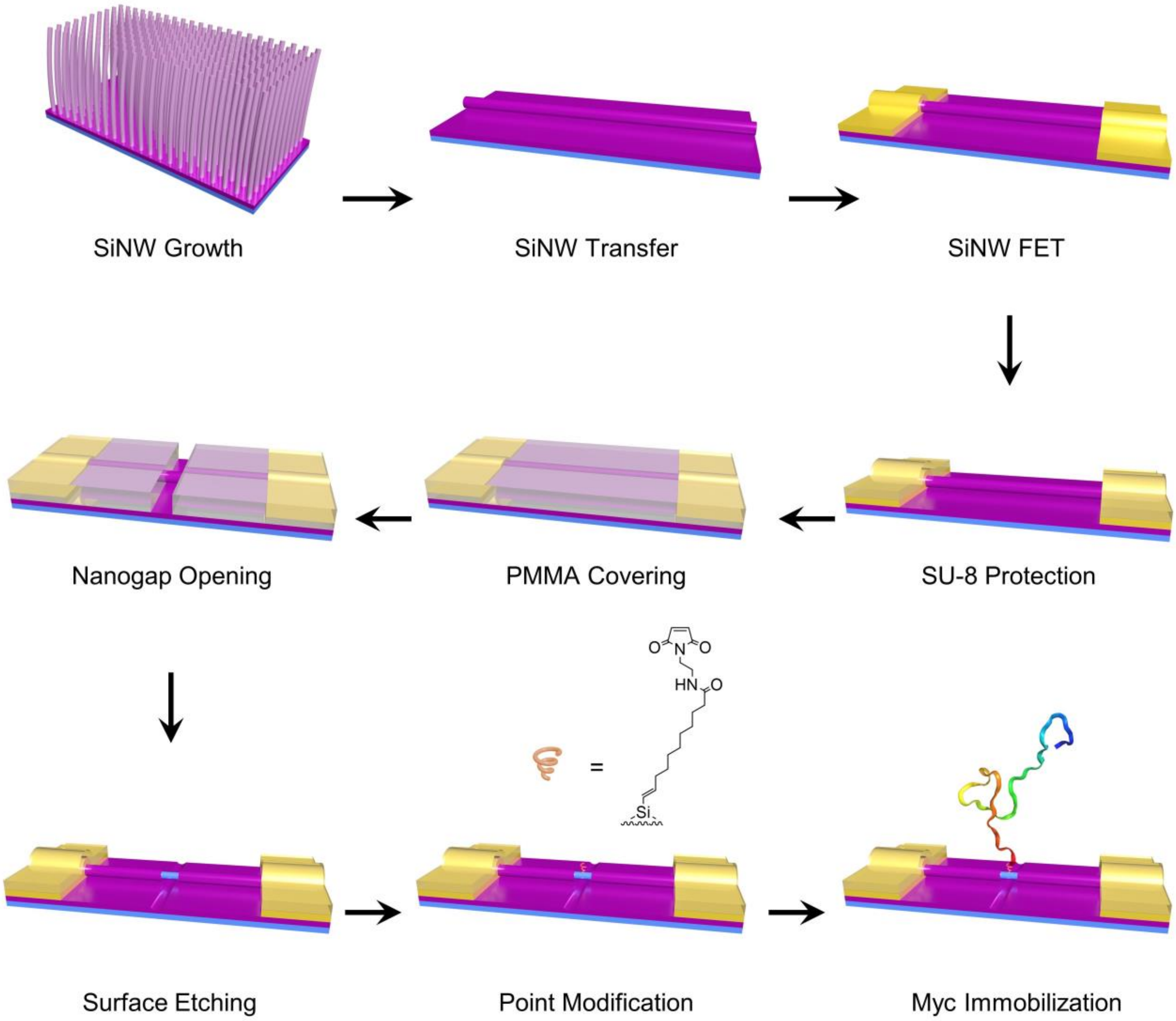

Supplementary Fig. 13 | Fabrication flowchart of single-peptide modified device.

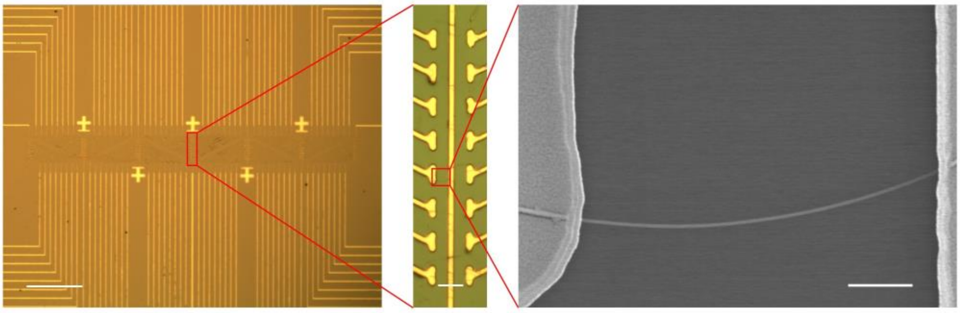

Supplementary Fig. 14 | Characterization of SiNW-FET device arrays. Left and medium images were taken by Nikon Eclipse LV 100 under 20x and 50x objectives. The right SEM image was generated by Hitachi S-4800. The scale bars from left to right are 300 µm, 10 µm and 1 µm, respectively. Experiments were repeatedly conducted over three times with similar results and one representative image was chosen for analysis.

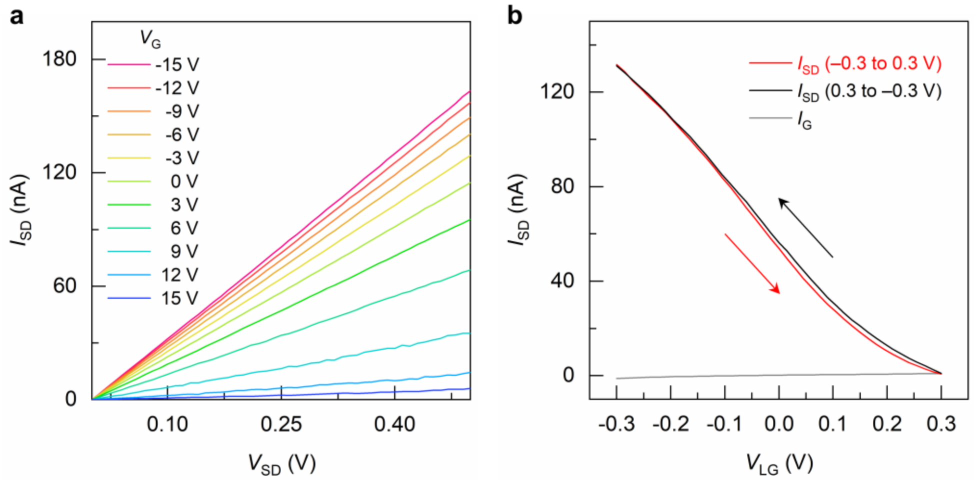

Supplementary Fig. 15 | Electrical characterization of SiNW-FETs. a, Output curves of a *p*-type SiNW-FET. V_{SD} scanned from 0 to 0.5 V at a certain V_G (from –15 to 15 V with an interval of 3 V). **b,** Transfer curve of a *p*-type SiNW-FET. The device was measured under liquid gate by using a platinum probe as the third electrode in a PBS buffer solution (red line: from –0.3 to 0.3 V, black line: from 0.3 to –0.3 V, grey line: gate leakage current).

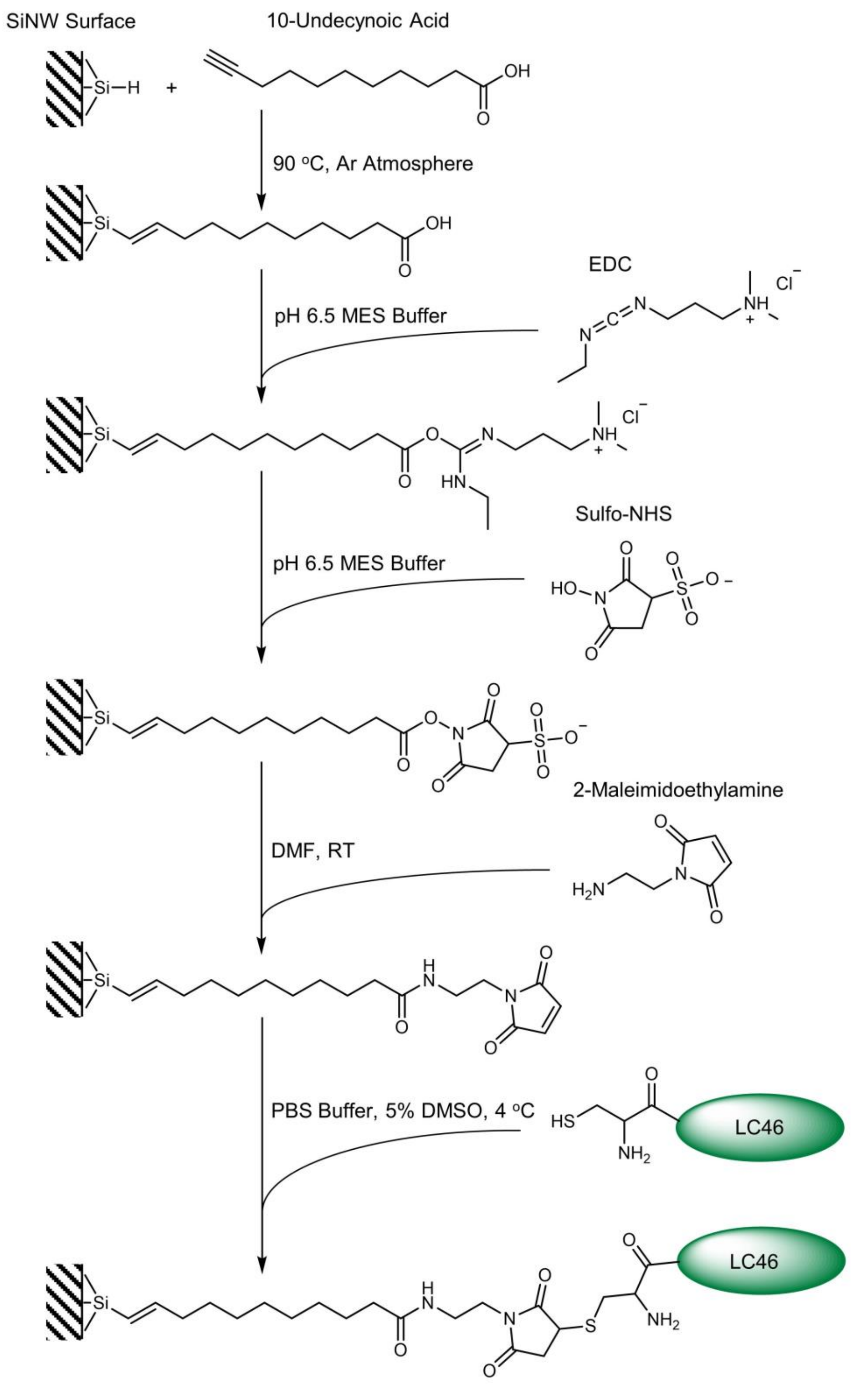

Supplementary Fig. 16 | Schematic of the point modification procedure after wet-etching.

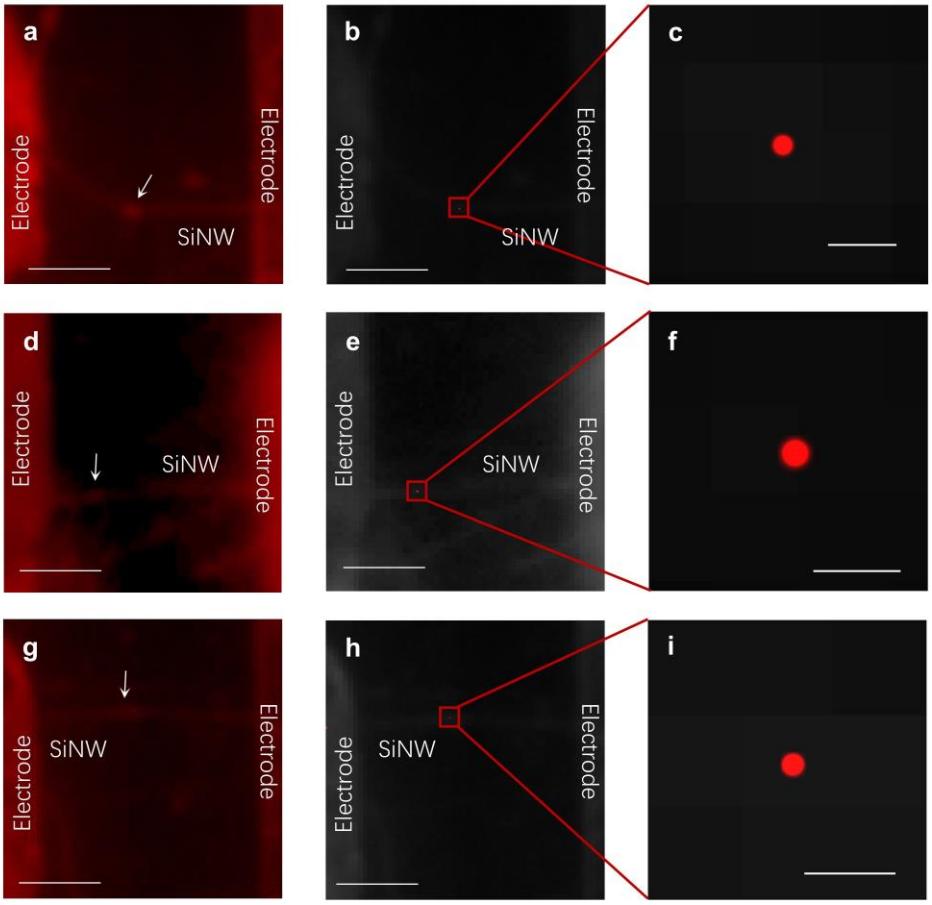

Supplementary Fig. 17 | Fluorescence characterization of single-Myc-modified devices. a, d and g, Images of single-LC46-modified devices in the dark field. The photos were taken under the excitation light of 480 nm wavelength. **b, e and h,** Stochastic optical reconstructions of 5000 photos for each device. **c, f and i,** Magnification of the reconstruction spots in the medium column. Scale bars from left to right are 2 μm, 2 μm and 160 nm.

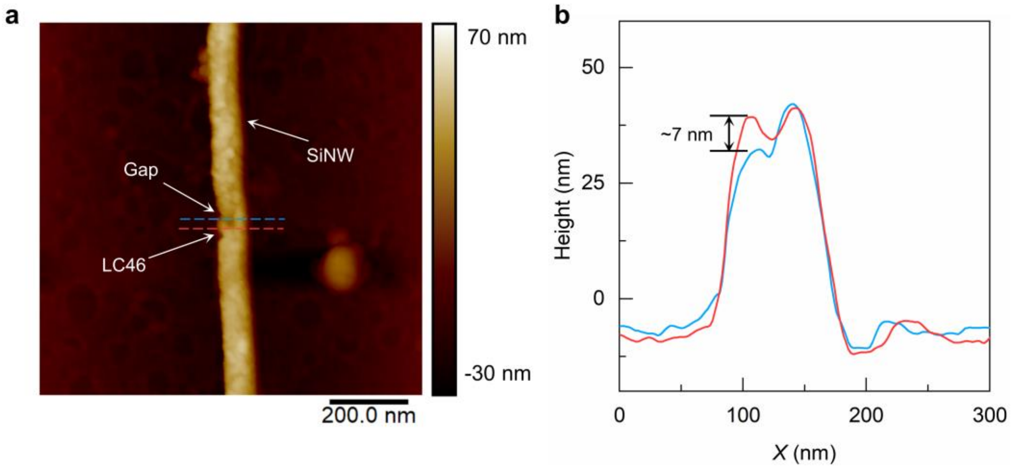

Supplementary Fig. 18 | AFM image of a single-LC46 modified SiNW-FET device. a) AFM image of a SiNW device, where a single LC46 was attached on the surface of a SiNW. **b**) The height distribution of different sections in the AFM image (blue for bare SiNW and red for the locus of protein attachment). The difference of the height is about 7 nm, which is consistent with the size of LC46 (~5.3 nm) and the molecule linkage (~1.7 nm). Experiments were repeatedly conducted three times with similar results and one representative image was chosen for analysis.

Supplementary Note 3: Real-time current measurements and dynamic analysis

Statistical Analysis: The current data were recorded with a combination of HF2LI Lock-in Amplifier (Zurich Instruments) and DL1211 preamplifier at a sampling rate of 57.6 or 28.8 kHz. The raw data with a high bandwidth (10 kHz) was then used to reduce the signal noise of the circuit by a low-pass Butterworth filter at a frequency of 5 kHz. The additional filtered processes were carried out by MATLAB 2016b. The QuB 2.0.0.32 software was then used to idealize the filtered data based on the hidden Markov model[7]. The dwell time of each signal event and the number of total events were extracted after the idealization. The extracted data were then analyzed with Origin 9.0. The dwell time was then fitted to a single-exponential decay function and the average dwell time was generated. Data are presented as mean ± standard deviation (SD). Populations of certain species in concentration-dependent experiments were fitted by the Hill Equation (Supplementary Eq. 1). The α represented the population ratio of binding species, $[L]$ for ligand concentration and K_D for dissociation constant.

$$\alpha = \frac{[L]^n}{K_D + [L]^n} \qquad \text{(Supplementary Eq. 1)}$$

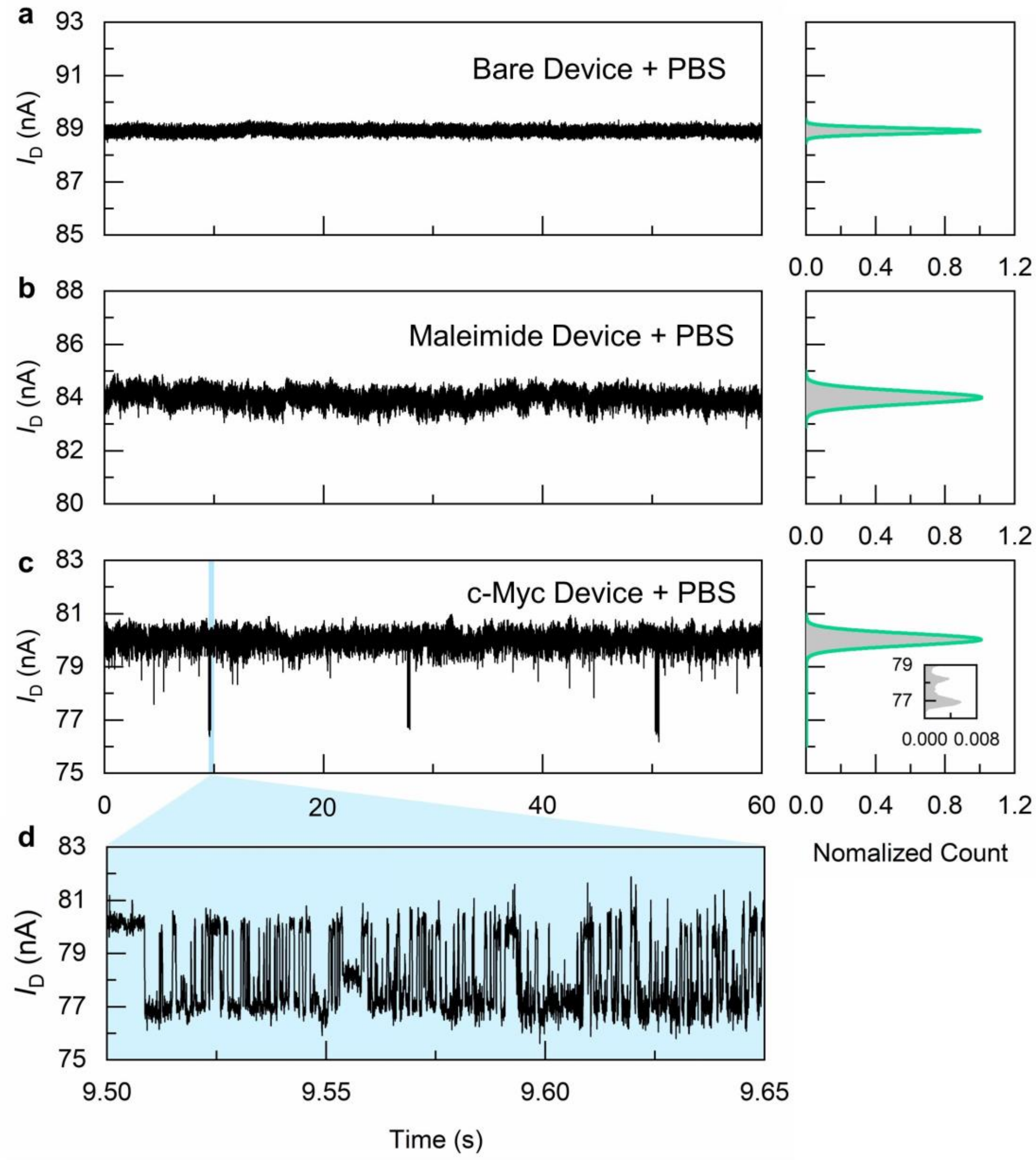

Supplementary Fig. 19 | Real-time measurements of control devices and experimental devices. a, Real-time trajectory of a bare SiNW-device. **b**, Real-time trajectory of a maleimide-modified device in blank buffer solution (0.01× PBS buffer, pH = 7.4, 5% DMSO). **c**, Real-time trajectory of a single-Myc modified device in blank buffer solution (0.01× PBS buffer, pH = 7.4, 5% DMSO). **d**, Magnified view of the data marked with blue in **c**.

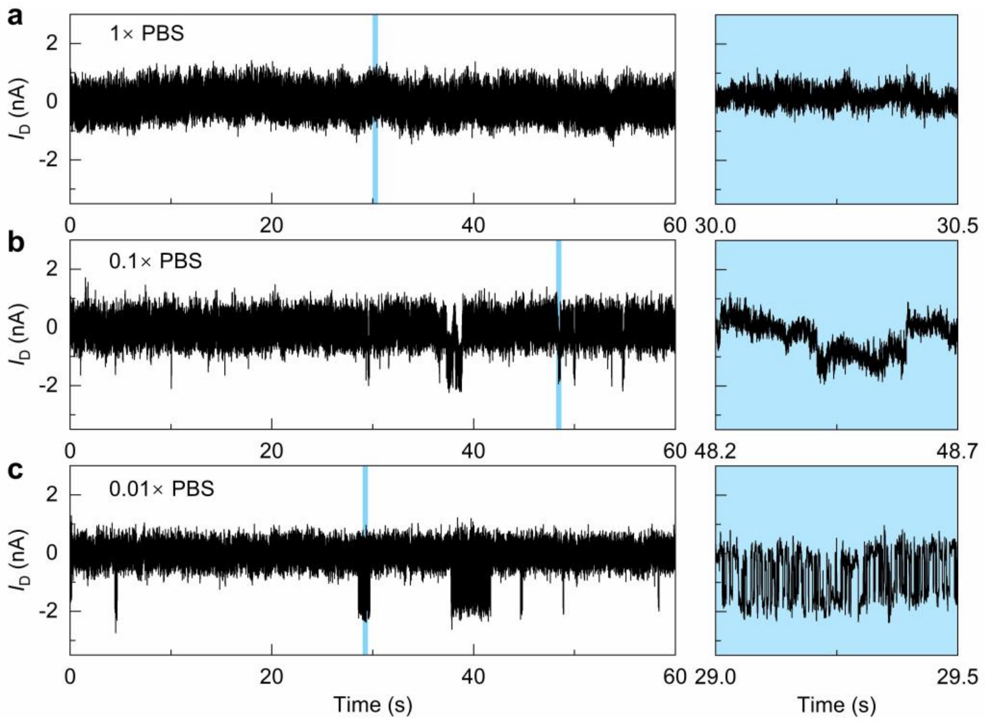

Supplementary Fig. 20 | PBS concentration-dependent experiments. a–c, Real-time current trajectories of a single-Myc modified device in different PBS solutions (PBS buffer [8g/L NaCl, 0.2 g/L KCl, 1.44 g/L Na_2HPO_4 and 0.24 g/L KH_2PO_4], pH = 7.4, 5% DMSO): 1× PBS (**a**), 0.1× PBS (**b**), 0.01× PBS (**c**). The right column is the 60-s current data, and the left column is the magnified view of the selected area.

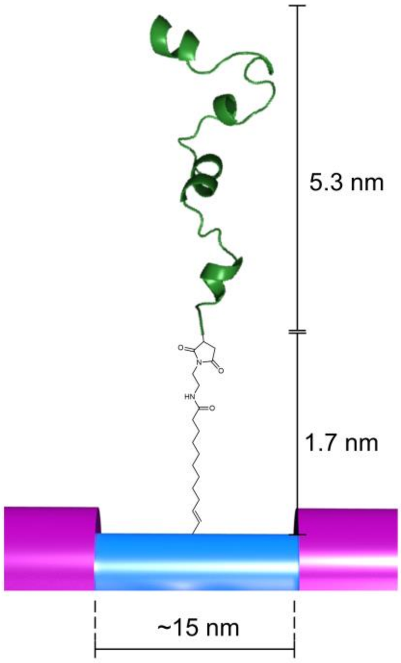

Supplementary Fig. 21 | Dimension information of a LC46 modified device. Prediction of the molecular length of apo-LC46 and molecule linkage**.**

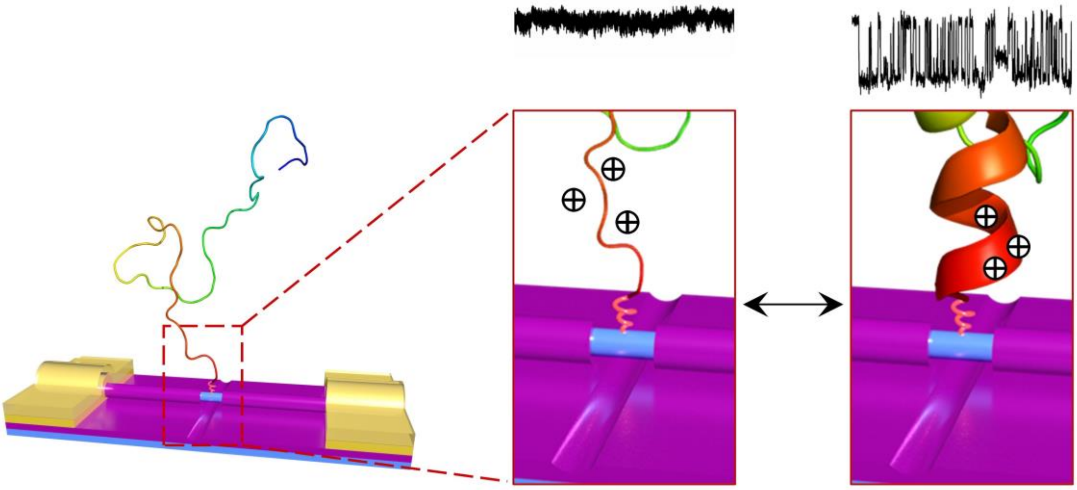

Supplementary Fig. 22 | Sensing principle of a *p*-type SiNW-FET device with c-Myc modification.

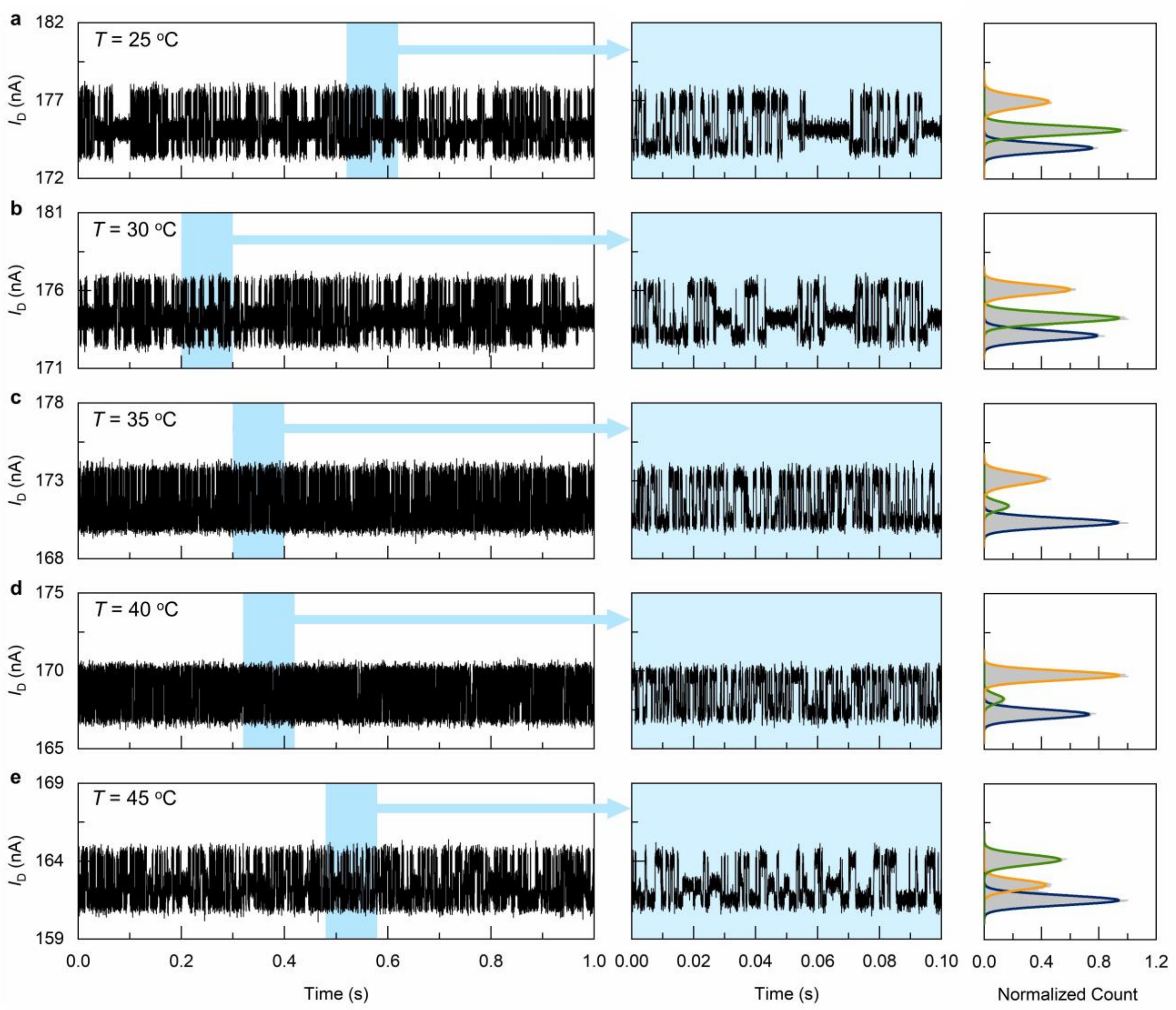

Supplementary Fig. 23 | Temperature-dependent experiments of the c-Myc IDR folding process. a–e, Real-time current trajectories of a single-Myc modified device in the blank buffer solution (0.01 × PBS buffer, pH = 7.4, 5% DMSO) at different temperatures: 25 ℃ (**a**), 30 ℃ (**b**), 35 ℃ (**c**), 40 ℃ (**d**), and 45 ℃ (**e**). The right column is the 1 s current data, the middle column is the magnified view of 0.1s marked with blue areas, and the left column is the statistical histogram of the current data. The current data is collected with ~17 μs temporal resolution.

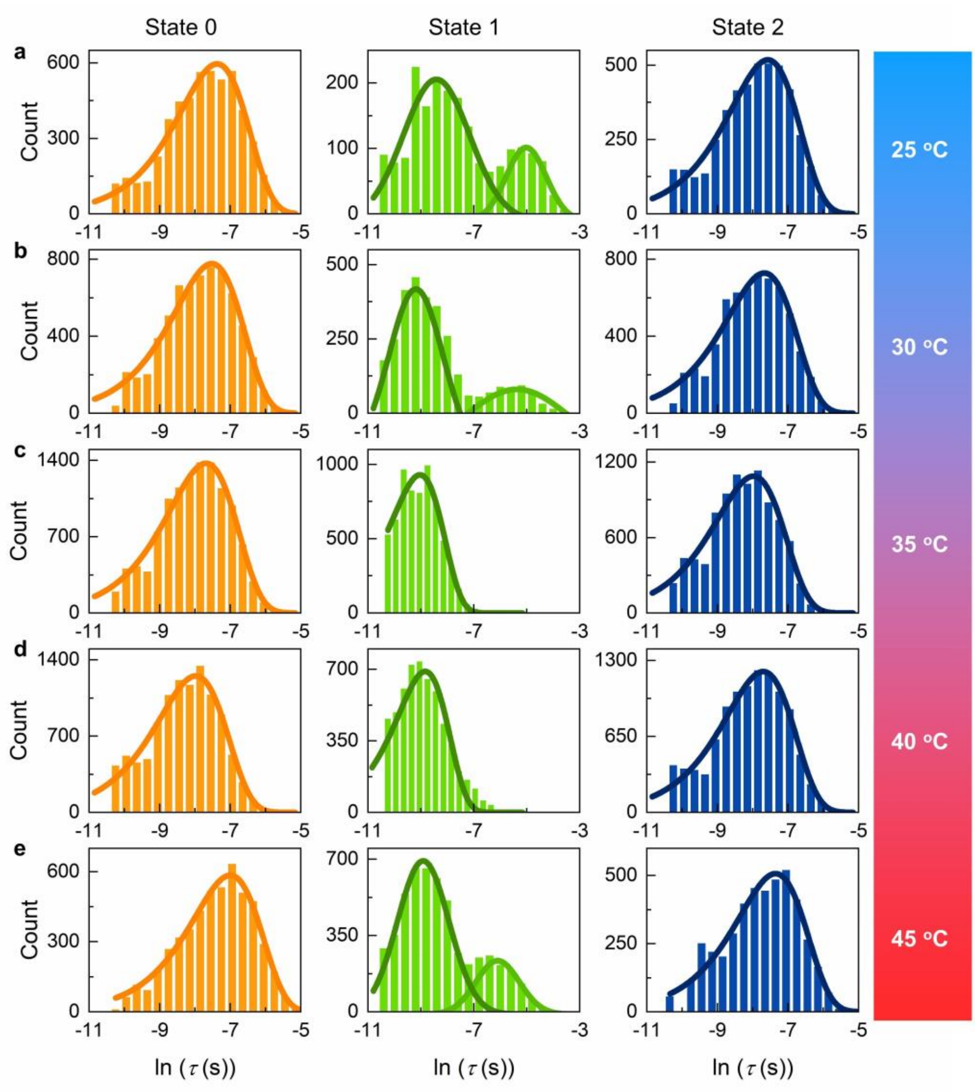

Supplementary Fig. 24 | The dwell-time distributions of different current states at different temperatures. a–e, The dwell-time distributions of three self-folding conformational intermediates at different temperatures: 25 °C (**a**), 30 °C (**b**), 35 °C (**c**), 40 °C (**d**), and 45 °C (**e**). The left, middle and right columns are the dwell time distributions of high, medium and low current states (0, 1 and 2 states), respectively.

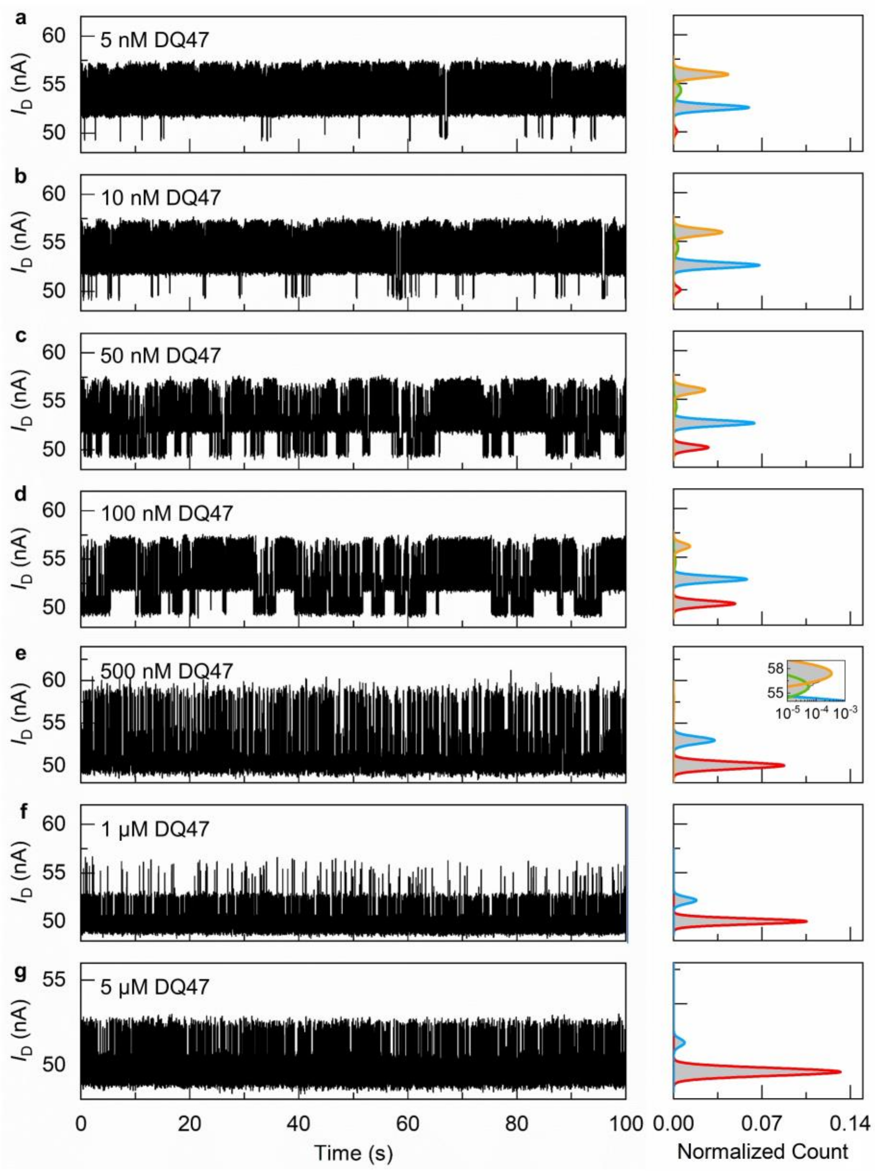

Supplementary Fig. 25 | Max concentration-dependent experiments at pH = 7.4. a–g, Real-time current trajectories of a single-Myc modified device in the solution (0.01× PBS buffer, pH = 7.4, 5% DMSO) with different Max concentrations: 5 nM (**a**), 10 nM (**b**), 50 nM (**c**), 100 nM (**d**), 500 nM (**e**), 1 μM (**f**), and 5 μM (**g**). The right column is the 100 s current data, and the left column is the statistical histogram of the current data. The current data is collected with ~17 μs temporal resolution.

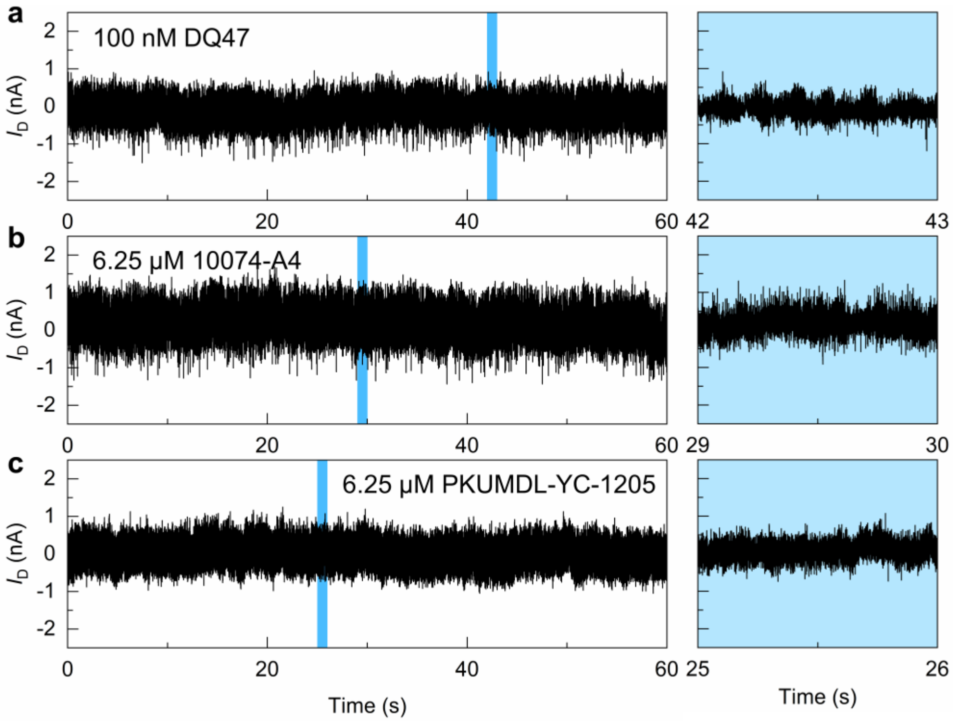

Supplementary Fig. 26 | Control experiments using molecule-linkage devices without LC46 modification. a–c, Real-time current trajectories of a molecule-linkage modified device in different solutions (PBS buffer [8g/L NaCl, 0.2 g/L KCl, 1.44 g/L Na_2HPO_4 and 0.24 g/L KH_2PO_4], pH = 7.4, 5% DMSO): 100 nM DQ47 (**a**), 6.25 μM 10074-A4 (**b**), 6.25 μM PKUMDL-YC-1205 (**c**). The right column is the 60 s current data, and the left column is the magnified view of the selected area.

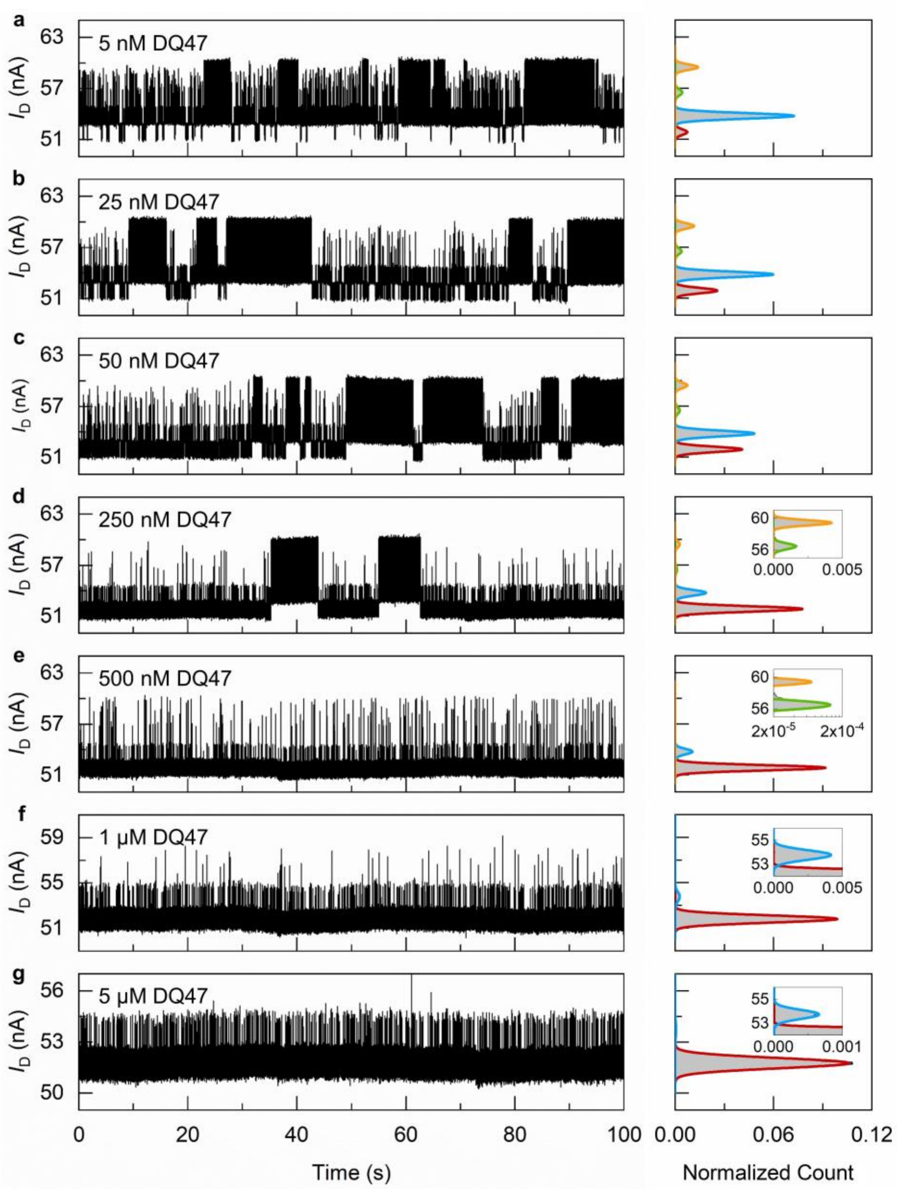

Supplementary Fig. 27 | Max concentration-dependent experiments at pH = 6.5. a–g, Real-time current trajectories of a single-Myc modified device in the solution (0.01× PBS buffer, pH = 6.5, 5% DMSO) with different DQ47 concentrations: 5 nM (**a**), 10 nM (**b**), 50 nM (**c**), 100 nM (**d**), 500 nM (**e**), 1 μM (**f**), and 5 μM (**g**). The right column is the 100 s current data, and the left column is the statistical histogram of the current data.

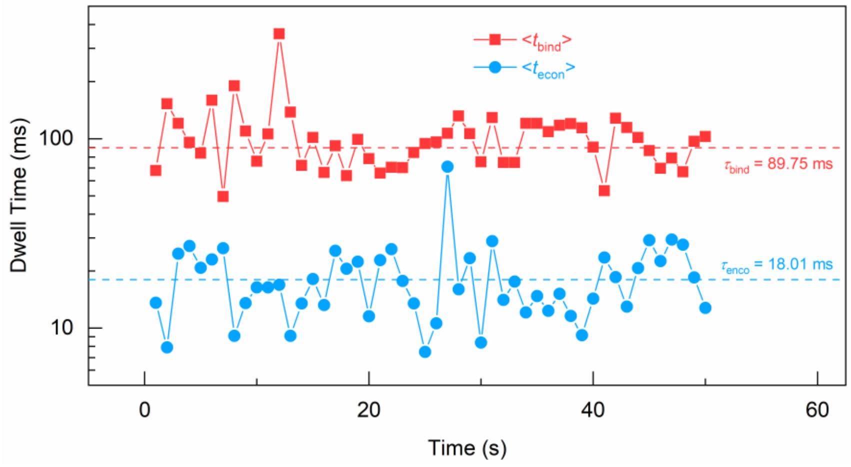

Supplementary Fig. 28 | Variation in the dwell-time mean values ⟨*t*⟩ of DQ47-LC46 binding kinetics. These values were arithmetic mean values calculated from dwell times within indicated 1-s data over a 50-second interval at 37 ℃ with 1 μM DQ47. The corresponding τ derived from the exponential fit of each state is indicated by dashed lines (red for binding state and blue for encounter state through dissociation).

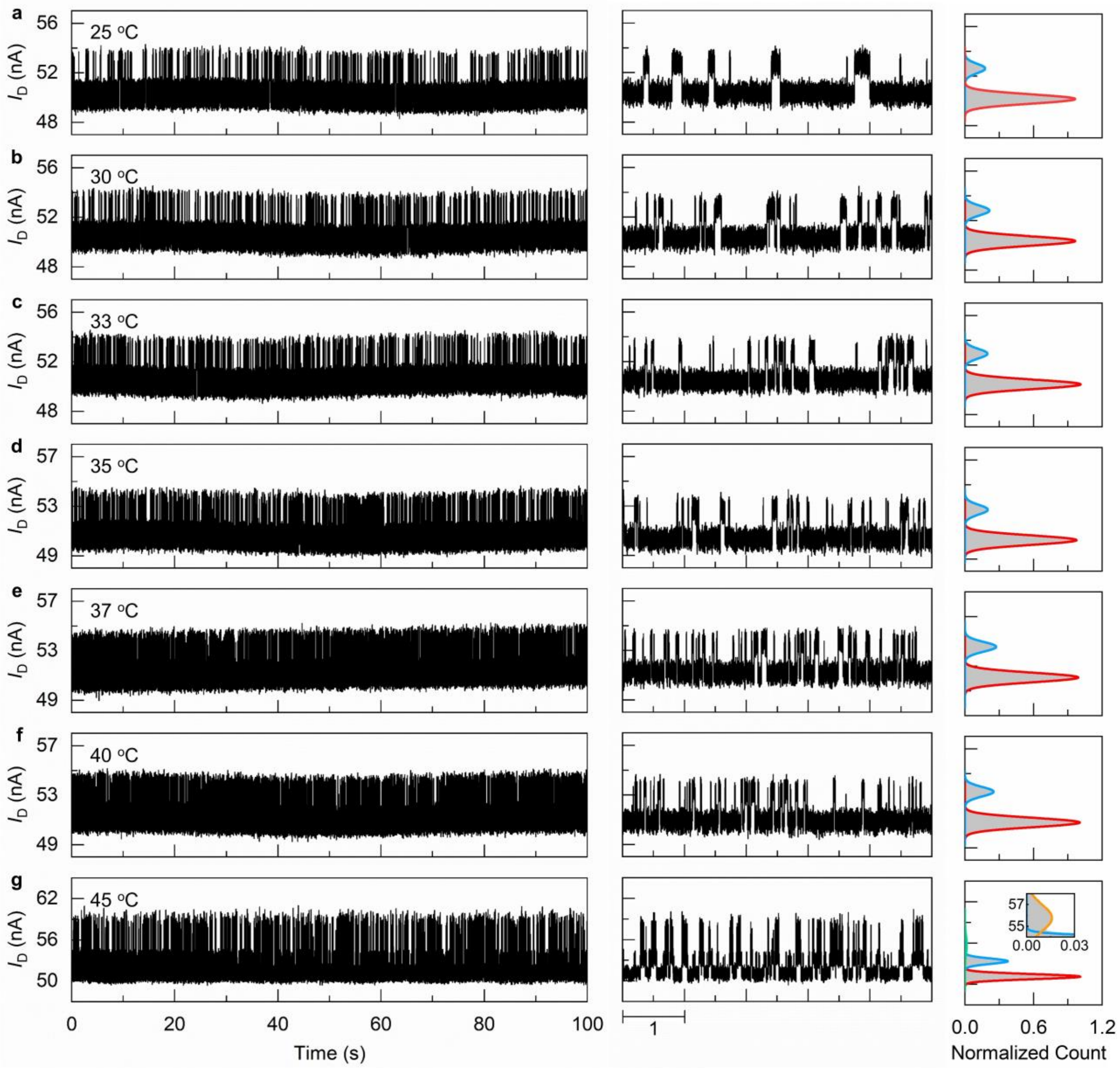

Supplementary Fig. 29 | Temperature-dependent experiments of the Myc-Max binding process at pH = 7.4. a–g, Real-time current trajectories of a single-Myc modified device in 1 μM DQ47 solution (0.01× PBS buffer, pH 7.4, 5% DMSO) at different temperatures: 25 ℃ (**a**), 30 ℃ (**b**), 33 ℃ (**c**), 35 ℃ (**d**), 37 ℃ (**e**), 40 ℃ (**f**), and 45 ℃ (**g**). The right column is the 1 s current data, the middle column is the magnified view of 0.1 s data, and the left column is the statistical histogram of the current data.

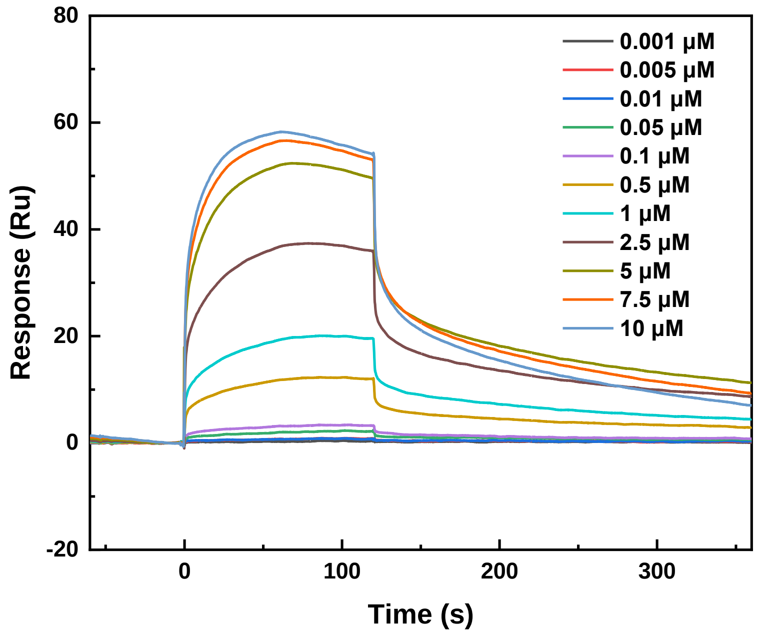

Supplementary Fig. 30 | Surface plasmon resonance assay of Myc with Max peptide. The association and dissociation curves of DQ47 at concentrations of 0.001–10 μM. The K_D value was 351 ± 28 nM based on kinetic fitting.

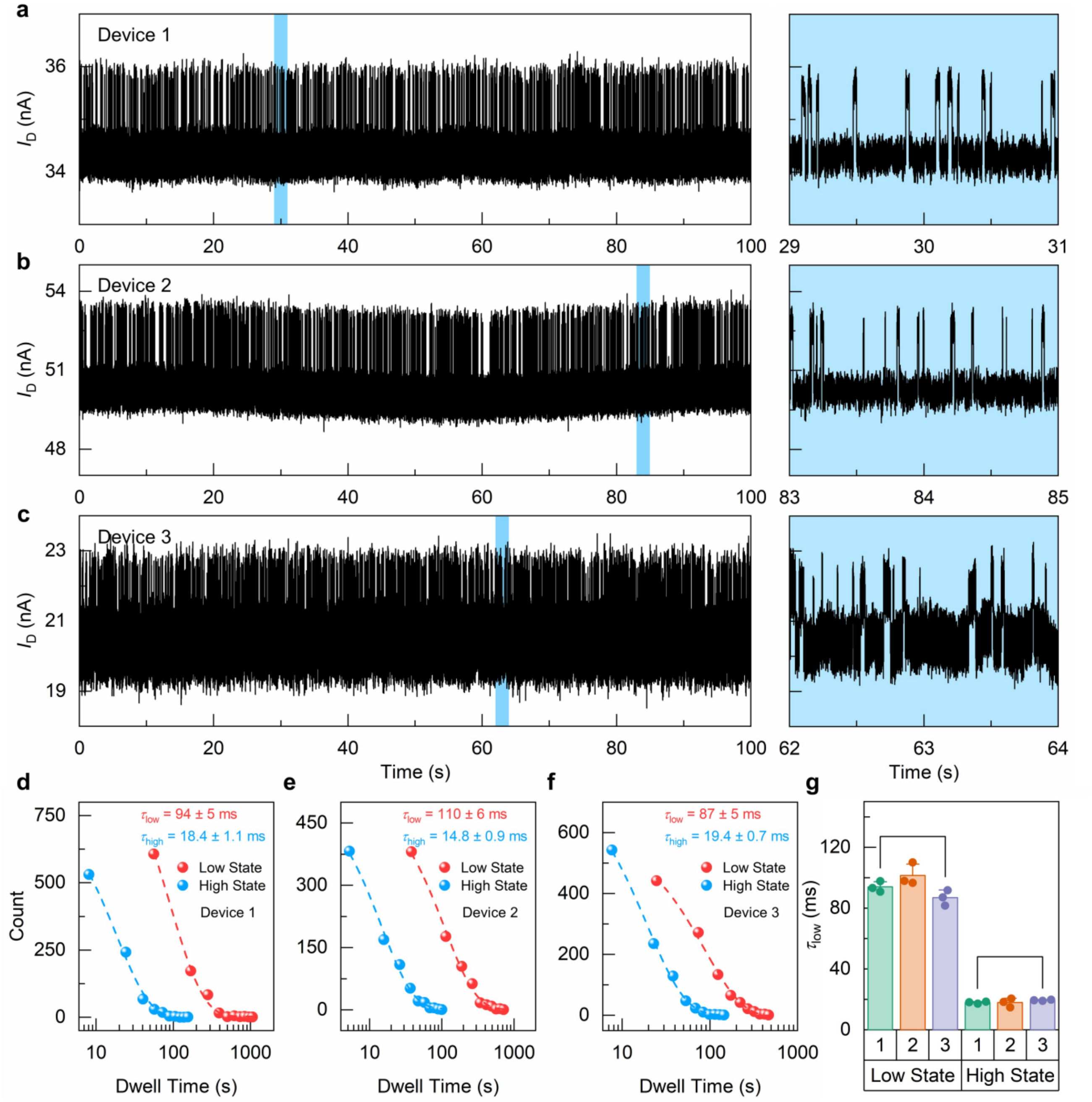

Supplementary Fig. 31 | Experiments measuring different devices in 1 μM DQ47 solution at 37 ℃. a–c, Real-time current trajectories from different devices in 1 μM DQ47 solution at 37 ºC (0.01 × PBS [8g/L NaCl, 0.2 g/L KCl, 1.44 g/L Na_2HPO_4 and 0.24 g/L KH_2PO_4], pH = 7.4, 5% DMSO): Device 1 (**a**), Device 2 (**b**), and Device 3 (**c**). The right column is the 100 s current data, and the left column is the magnified view of the selected area. **d–f,** Dwell-time distributions of the Myc-Max binding process from different devices: Device 1 (**d**), Device 2 (**e**), and Device 3 (**f**). **g**, Average dwell-time distributions from 3 current trajectories with different devices (green for Device 1, orange for Device 2, and purple for Device 3). The results from different devices are not significantly different for individual current states at 0.05 level from the one-way ANVOA analysis.

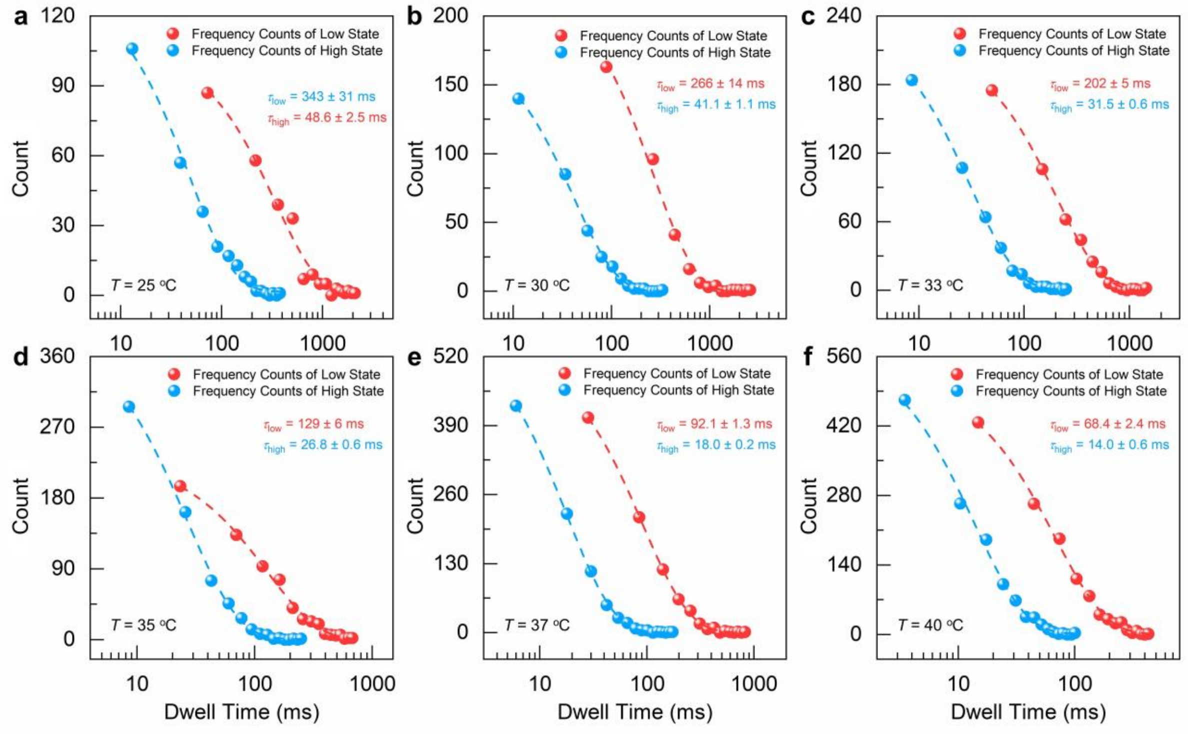

Supplementary Fig. 32 | Dwell-time distributions of the Myc-Max binding process at different temperatures. a–g, Dwell-time distributions of the Myc-Max binding process in 1 μM Max solution (0.01× PBS buffer, pH = 7.4, 5% DMSO) at different temperatures: 25 °C (**a**), 30 °C (**b**), 33 °C (**c**), 35 °C (**d**), 37 °C (**e**), 40 °C (**f**), and 45 °C (**g**). Dissociation states are shown in red and binding states are shown in blue. The dwell-time distributions can be fitted by a single exponential function to obtain the averaged dwell times at different temperatures.

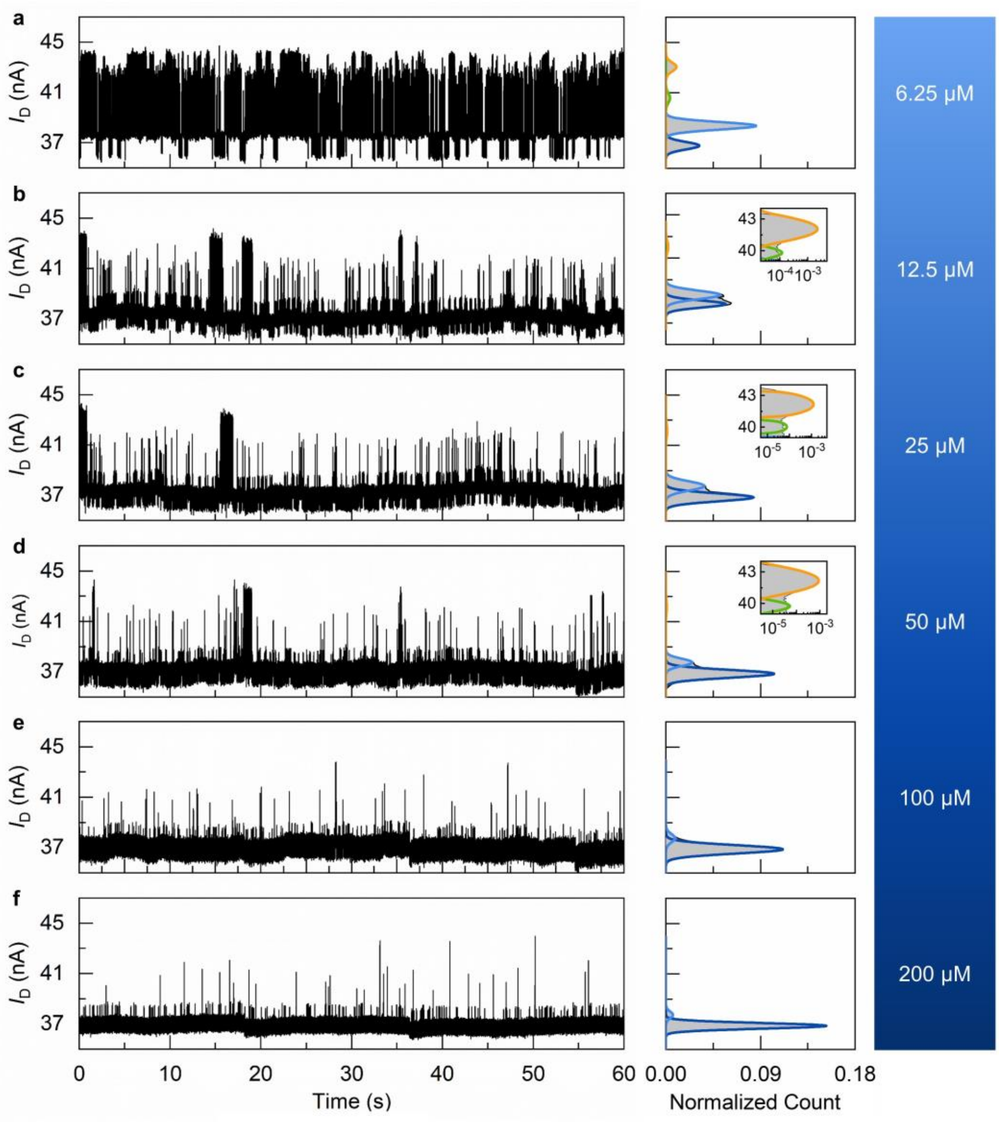

Supplementary Fig. 33 | 10074-A4 concentration-dependent experiments under the physiological condition. a–f, Real-time current trajectories of a single-Myc modified device in the solution (0.01× PBS buffer, pH = 7.4, 5% DMSO) with different 10074-A4 concentrations: 6.25 μM (**a**), 12.5 μM (**b**), 25 μM (**c**), 50 μM (**d**), 100 μM (**e**), and 200 μM (**f**). The right column is the 60 s current data, and the left column is the statistical histogram of the current data.

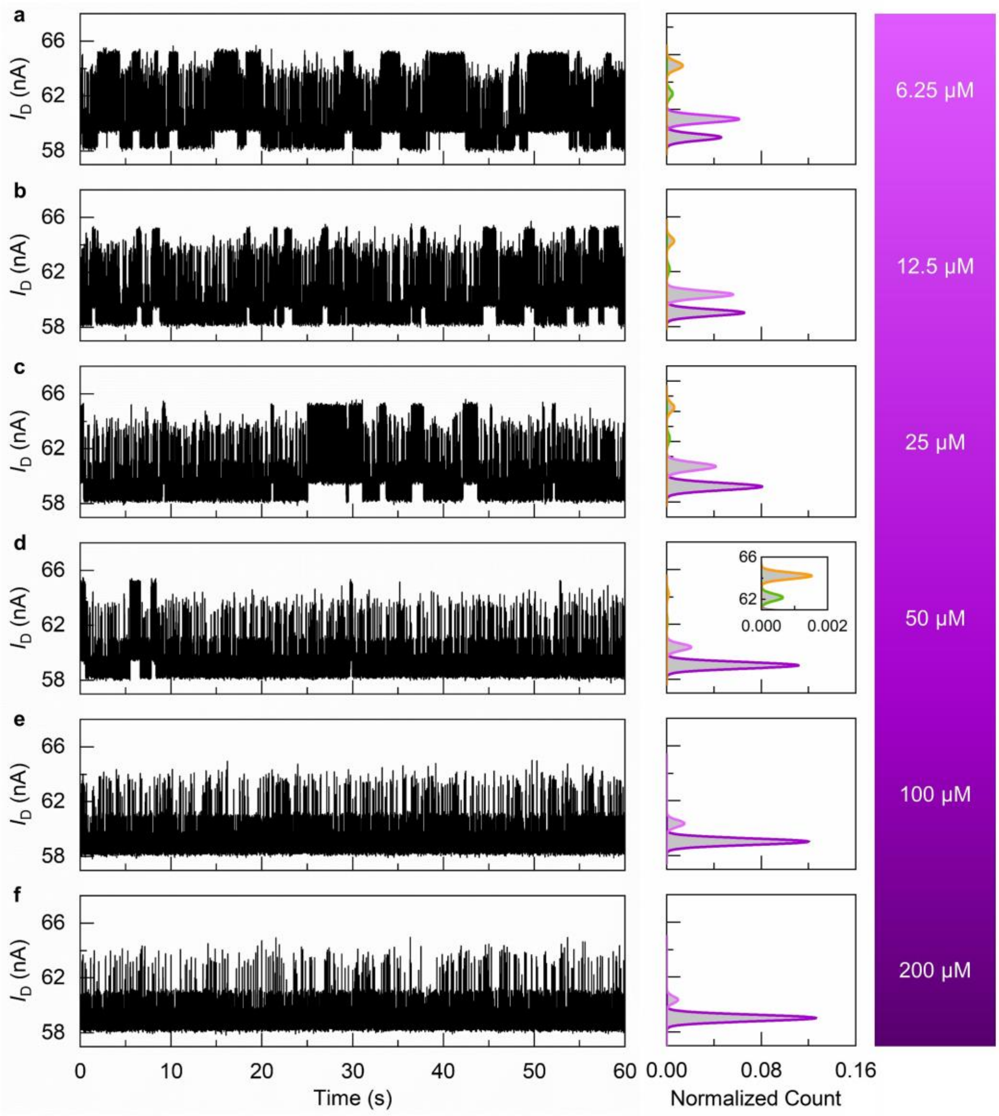

Supplementary Fig. 34 | PKUMDL-YC-1205 concentration-dependent experiments under the physiological condition. A–f, Real-time current trajectories of a single-Myc modified device in the solution (0.01× PBS buffer, pH = 7.4, 5% DMSO) with different 10074-A4 concentrations: 6.25 μM (**a**), 12.5 μM (**b**), 25 μM (**c**), 50 μM (**d**), 100 μM (**e**), and 200 μM (**f**). The right column is the 60 s current data, and the left column is the statistical histogram of the current data.

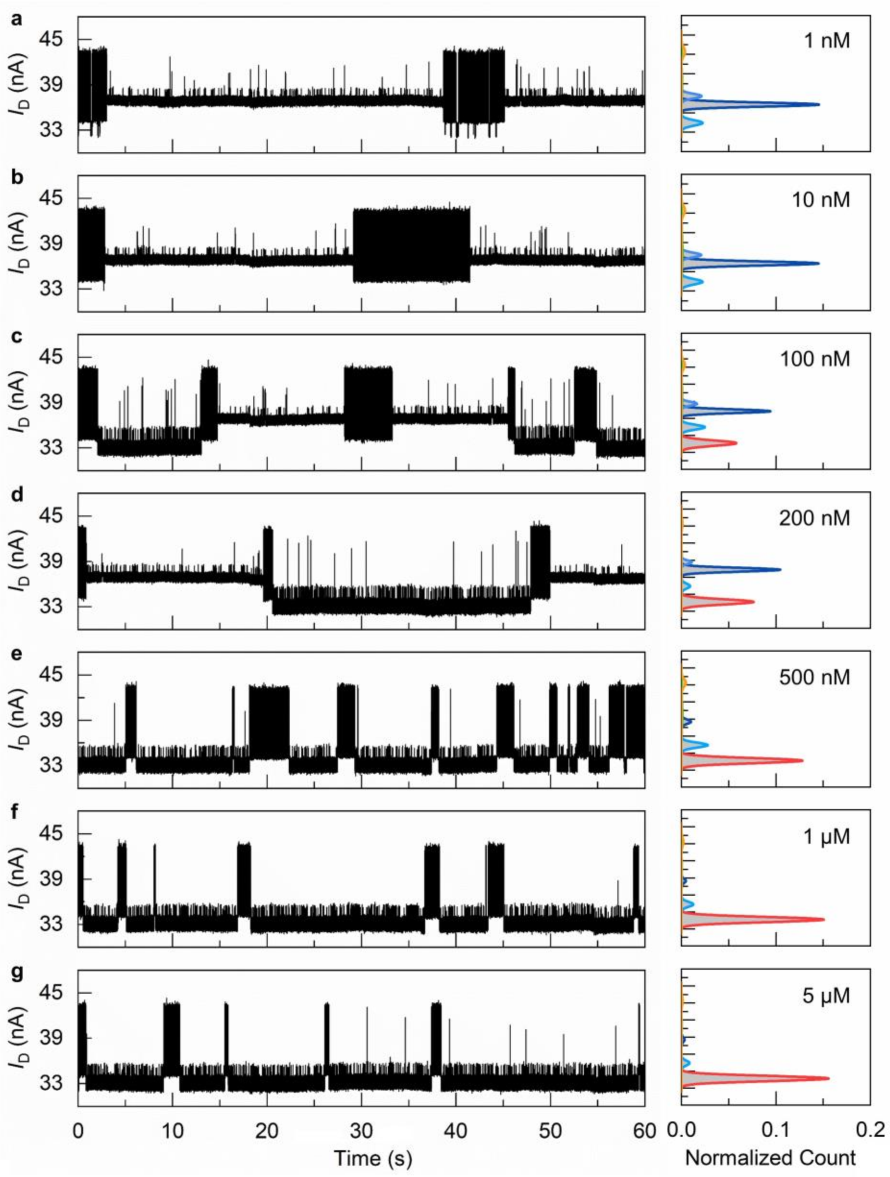

Supplementary Fig. 35 | Max concentration dependent-experiments in competition with 100 μM 10074-A4. a–g, Real-time current trajectories of a single-Myc modified device in the 100 μM 10074-A4 solution (0.01× PBS buffer, pH = 7.4, 5% DMSO) with different DQ47 concentrations: 5 nM (**a**), 10 nM (**b**), 50 nM (**c**), 100 nM (**d**), 500 nM (**e**), 1 μM (**f**), and 5 μM (**g**). The right column is the 100 s current data, and the left column is the statistical histogram of the current data.

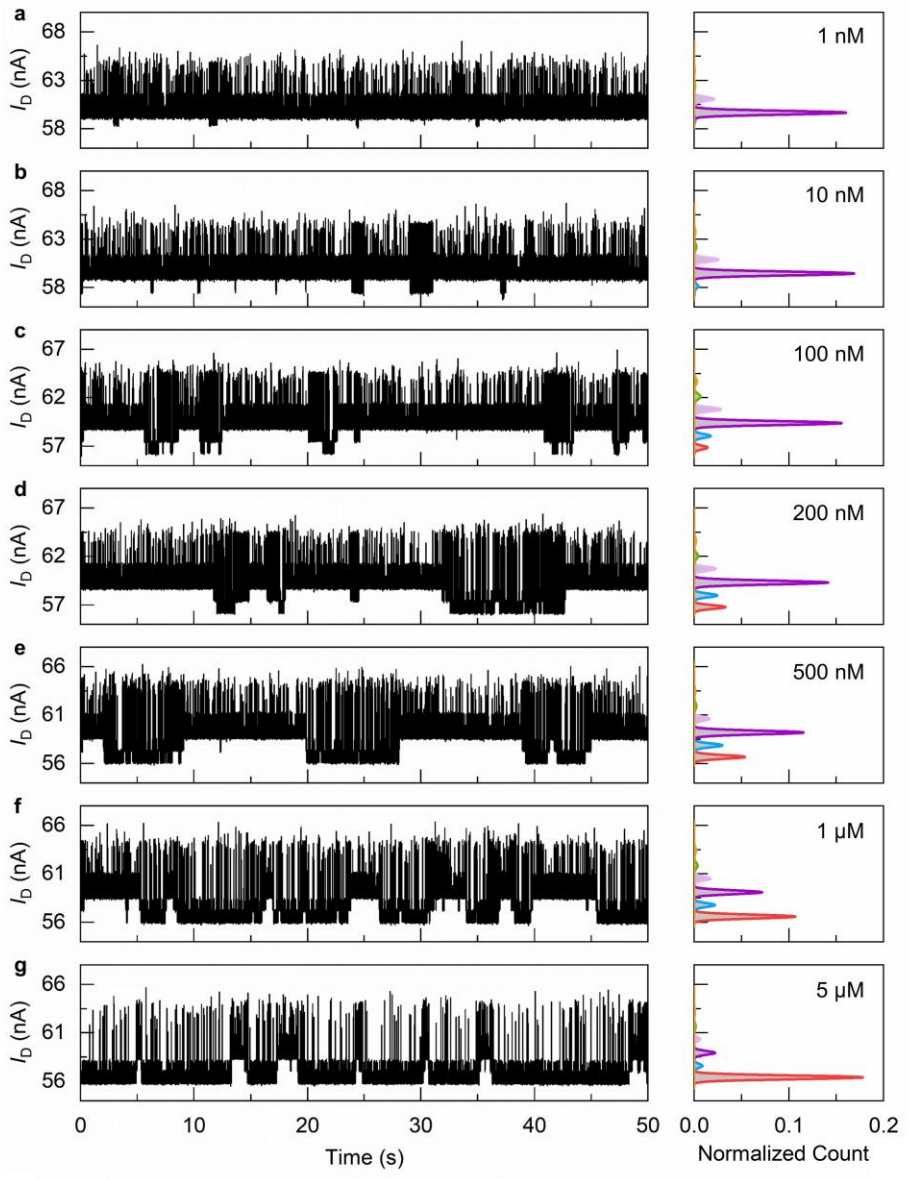

Supplementary Fig. 36 | Max concentration-dependent experiments in competition with 100 μM PKUMDL-YC-1205. a–g, Real-time current trajectories of a single-Myc modified device in the 100 μM PKUMDL-YC-1205 solution (0.01× PBS buffer, pH = 7.4, 5% DMSO) with different DQ47 concentrations: 5 nM (**a**), 10 nM (**b**), 50 nM (**c**), 100 nM (**d**), 500 nM (**e**), 1 μM (**f**), and 5 μM (**g**). The right column is the 100 s current data, and the left column is the statistical histogram of the current data.

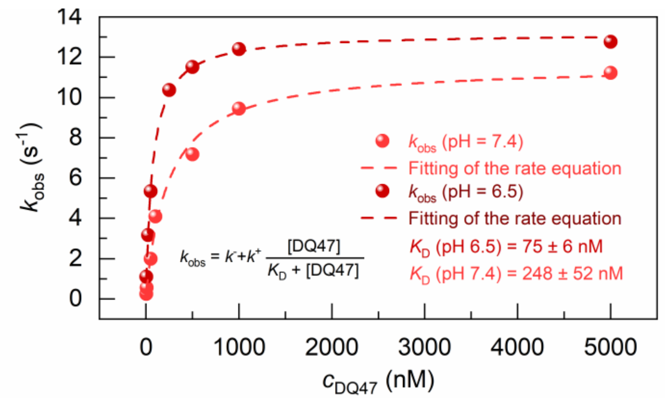

Fig. 37 | k_{obs} at different DQ47 concentrations and different pHs. The k_{obs} is regarded as the number of occurrences per second of the fully folded binding complex. The k_{obs} can be fitted by Supplementary Eq. 2 derived from a two-step induce-fit model[8]. Red for pH 7.4 and dark red for pH 6.5.

$$k_{obs} = k^- + k^+ \frac{[L]}{K_D + [L]} \quad \text{(Supplementary Eq. 2)}$$

Supplementary Note 4: Supplementary Tables

Supplementary Table 1. Debye lengths of different PBS concentrations.

PBS pH 7.4 (5% DMSO)	λ_D (nm)	ionic strength
1×	0.72	143 mM
0.1×	2.4	14 mM
0.01×	7.5	1.4 mM

The Debye lengths of different PBS concentrations can be calculated by Supplementary Eq. 3 as below.

$$\lambda_D = \sqrt{\frac{\varepsilon k_B T}{q^2 c}} \qquad \text{(Supplementary Eq. 3)}$$

where ε is the dielectric permittivity of the media, k_B is Boltzmann's constant, T is the temperature, q is the electron charge, and c represents the ionic strength of the electrolyte[9].

Supplementary Table 2. Binding affinity of Myc and Max at different PBS concentrations.

PBS pH 7.4 (5% DMSO)	K_D (nM)
1×	351 ± 28
0.01×*	374 ± 147

*0.01× PBS, 0.2% T20, 5% DMSO, pH 7.4

Supplementary Table 3. Averaged dwell times of four conformational ensembles at five different temperatures. The SDs are calculated from three different devices.

τ	T = 25 °C	T = 30 °C	T = 35 °C	T = 40 °C	T = 45 °C
τ_0 (ms)	0.643 ± 0.058	0.552 ± 0.076	0.454 ± 0.035	0.333 ± 0.083	0.872 ± 0.148
τ_1 (ms)	0.215 ± 0.061	0.111 ± 0.029	0.109 ± 0.027	0.122 ± 0.069	0.147 ± 0.033
τ_{1*} (ms)	6.486 ± 0.717[1]	4.556 ± 0.665[1]	-	-	2.283 ± 0.494[2]
τ_2 (ms)	0.541 ± 0.119	0.458 ± 0.044	0.342 ± 0.120	0.447 ± 0.065	0.670 ± 0.092

[1]State 1^*_{LT}

[2]State 1^*_{HT}

Supplementary Table 4. Averaged populations of LC46-DQ47 dimer at seven different concentrations of DQ47 under different pHs. The SDs are calculated from three different devices.

**pH = 6.5**		**pH = 7.4**	
c_{DQ47} **(nM)**	α_{bind} **(%)**	c_{DQ47} **(nM)**	α_{bind} **(%)**
5	6.7 ± 0.8	5	2.3 ± 0.5
25	24.0 ± 2.4	10	5.4 ± 1.1
50	37.5 ± 2.3	50	18.3 ± 3.1
250	74.4 ± 2.2	100	39.0 ± 5.2
500	86.2 ± 3.7	500	72.3 ± 2.8
1000	92.9 ± 3.2	1000	84.7 ± 1.5
5000	97.6 ± 1.7	5000	94.2 ± 3.5

Supplementary Table 5. Detailed kinetic and thermodynamic parameters at different temperatures in 1 μM DQ47 solution: The dwell times of binding and dissociation states (τ_{bind} and τ_{diss}), rate constants of binding and dissociation process (k_{bind} and k_{diss}), and the dissociation constants (K_D). The SDs are calculated from three different devices.

T (℃)	τ_{bind} (ms)	τ_{diss} (ms)	k_{diss} (s^{-1})	k_{bind} (s^{-1})	K_D (nM)
25	388 ± 109	50 ± 7	2.6 ± 0.7	20 ± 3	146 ± 27
30	283 ± 51	42 ± 14	3.5 ± 0.6	24 ± 8	155 ± 53
33	199 ± 18	34 ± 2	5.0 ± 0.5	29 ± 2	172 ± 17
35	129 ± 8	25 ± 2	7.8 ± 0.5	40 ± 3	196 ± 14
37	94 ± 21	18 ± 3	10.7 ± 2.4	56 ± 8	200 ± 48
40	69 ± 5	15 ± 1	14.6 ± 1.0	66 ± 5	223 ± 18

Supplementary Table 6. Averaged populations of LC46-inhibitor complex at different concentrations. The SDs are calculated from three different devices.

$c_{inhibitor}$ (μM)	$\alpha_{bind\text{-}10074\text{-}A4}$ (%)	$\alpha_{bind\text{-}PKUMDL\text{-}YC\text{-}1205}$ (%)
6.25	21.4 ± 2.0	29.6 ± 4.1
12.5	44.9 ± 2.2	43.8 ± 4.4
25	62.6 ± 2.0	62.3 ± 3.3
50	80.5 ± 1.1	78.2 ± 4.4
100	92.4 ± 2.8	87.9 ± 1.3
200	94.3 ± 1.9	92.5 ± 1.8

Supplementary Table 7. Averaged populations of LC46-DQ47 dimer at seven different concentrations of DQ47 in 100 μM inhibitor solution. The SDs are calculated from three different devices.

c_{DQ47} **(nM)**	α_{bind}**@10074-A4**	α_{bind}**@PKUMDL-YC-1205**
1	1.1 ± 0.4	0.06 ± 0.05
10	1.2 ± 0.4	0.1 ± 0.1
100	21.0 ± 1.2	7.3 ± 3.5
200	47.5 ± 2.7	13.8 ± 3.1
500	75.9 ± 2.4	28.7 ± 8.6
1000	91.2 ± 2.4	45.5 ± 8.2
5000	90.2 ± 3.1	81.8 ± 6.1

Supplementary Note 5: Supplementary References

[1] Patolsky, F., Zheng, G. F. & Lieber, C. M. Fabrication of silicon nanowire devices for ultrasensitive, label-free, real-time detection of biological and chemical species. *Nat. Protoc.* **1**, 1711-1724 (2006).

[2] Wang, J. D. *et al.* Point decoration of silicon nanowires: An approach toward single- molecule electrical detection. *Angew. Chem.Int. Ed.* **53**, 5038-5043 (2014).

[3] Li, J. *et al.* Direct measurement of single-molecule adenosine triphosphatase hydrolysis dynamics. *ACS Nano* **11**, 12789-12795 (2017).

[4] Liu, W. *et al.* Complete mapping of DNA-protein interactions at the single-molecule level. *Adv. Sci.* **8**, 2101383 (2021).

[5] Yin, D. *et al.* Direct mechano-sliding transfer of chemical vapor deposition grown silicon nanowires for nanoscale electronic devices. *J. Mater. Chem. C* **10**, 469-475 (2022).

[6] He, G., Li, J., Ci, H., Qi, C. & Guo, X. F. Direct measurement of single-molecule DNA hybridization dynamics with single-base resolution. *Angew. Chem.Int. Ed.* **55**, 9036-9040 (2016).

[7] Nicolai, C. & Sachs, F. Solving ion channel kinetics with the QuB software. *Biophys. Rev. & Lett.* **8**, 191-211 (2013).

[8] Vogt, A. D. & Di Cera, E. Conformational selection or induced fit? A critical appraisal of the kinetic mechanism. *Biochemistry* **51**, 5894-5902 (2012).

[9] Elnathan, R. *et al.* Biorecognition Layer Engineering: Overcoming Screening Limitations of Nanowire-Based FET Devices. *Nano Lett.* **12**, 5245-5254 (2012).
