# Supplementary material for: Visualizing single-molecule conformational transition and binding dynamics of intrinsically disordered proteins
Source: Nat Commun. 2023 Aug 25;14:5203. doi: 10.1038/s41467-023-41018-x (PMC10457384; doi:10.1038/s41467-023-41018-x)
Supplement: Supplementary file 3 — Reporting Summary [file 41467_2023_41018_MOESM3_ESM.pdf]

Corresponding author(s): Qi Sun, Luhua Lai, and Xuefeng Guo

Last updated by author(s): Jul 28, 2023

Reporting Summary

Nature Portfolio wishes to improve the reproducibility of the work that we publish. This form provides structure for consistency and transparency in reporting. For further information on Nature Portfolio policies, see our Editorial Policies and the Editorial Policy Checklist.

Statistics

For all statistical analyses, confirm that the following items are present in the figure legend, table legend, main text, or Methods section.

n/a	Confirmed	
☐	☒	The exact sample size (*n*) for each experimental group/condition, given as a discrete number and unit of measurement
☐	☒	A statement on whether measurements were taken from distinct samples or whether the same sample was measured repeatedly
☐	☒	The statistical test(s) used AND whether they are one- or two-sided<br>*Only common tests should be described solely by name; describe more complex techniques in the Methods section.*
☐	☒	A description of all covariates tested
☐	☒	A description of any assumptions or corrections, such as tests of normality and adjustment for multiple comparisons
☐	☒	A full description of the statistical parameters including central tendency (e.g. means) or other basic estimates (e.g. regression coefficient) AND variation (e.g. standard deviation) or associated estimates of uncertainty (e.g. confidence intervals)
☐	☒	For null hypothesis testing, the test statistic (e.g. *F*, *t*, *r*) with confidence intervals, effect sizes, degrees of freedom and *P* value noted<br>*Give P values as exact values whenever suitable.*
☒	☐	For Bayesian analysis, information on the choice of priors and Markov chain Monte Carlo settings
☐	☒	For hierarchical and complex designs, identification of the appropriate level for tests and full reporting of outcomes
☒	☐	Estimates of effect sizes (e.g. Cohen's *d*, Pearson's *r*), indicating how they were calculated

Our web collection on statistics for biologists contains articles on many of the points above.

Software and code

Policy information about availability of computer code

Data collection	The source-drain current through the selected SiNW device was amplified by a DL1211 preamplifier operating at 107 V/A gain and collected by the HF2LI Lock-in Amplifier with a bandwidth of 10 kHz low-pass filter at sampling rates of 28.8 or 7.2 kHz.
Data analysis	The I–t curves can be analysed by MATLAB 2016b, Origin 2019b, and QuB 2.0.0.32 software.

For manuscripts utilizing custom algorithms or software that are central to the research but not yet described in published literature, software must be made available to editors and reviewers. We strongly encourage code deposition in a community repository (e.g. GitHub). See the Nature Portfolio guidelines for submitting code & software for further information.

Data

Policy information about availability of data

All manuscripts must include a data availability statement. This statement should provide the following information, where applicable:

- Accession codes, unique identifiers, or web links for publicly available datasets
- A description of any restrictions on data availability
- For clinical datasets or third party data, please ensure that the statement adheres to our policy

All data that support the findings of this study are available within the main manuscript and the supplementary files. Source data are provided with this paper. All the datasets used in this work are available online from the Zenodo repository at https://zenodo.org/record/8188411. The protein structure (1NKP) used in this work is provided by RCSB PDB online (https://doi.org/10.2210/pdb1NKP/pdb).

Research involving human participants, their data, or biological material

Policy information about studies with human participants or human data. See also policy information about sex, gender (identity/presentation), and sexual orientation and race, ethnicity and racism.

Reporting on sex and gender	This study does not involve human research participants.
Reporting on race, ethnicity, or other socially relevant groupings	This study does not involve human research participants.
Population characteristics	See above.
Recruitment	Not applicable.
Ethics oversight	Not applicable.

Note that full information on the approval of the study protocol must also be provided in the manuscript.

Field-specific reporting

Please select the one below that is the best fit for your research. If you are not sure, read the appropriate sections before making your selection.

☐ Life sciences ☐ Behavioural & social sciences ☒ Ecological, evolutionary & environmental sciences

For a reference copy of the document with all sections, see nature.com/documents/nr-reporting-summary-flat.pdf

Ecological, evolutionary & environmental sciences study design

All studies must disclose on these points even when the disclosure is negative.

Study description	We established a point-functionalized silicon nanowire as a biosensor for real-time sampling of protein transient dynamics at single-molecule resolution. The parallel experiments were performed three times from three individual devices to ensure the reproducibility.
Research sample	A single intrinsically disordered protein was covalently conjugated to the silicon nanowire through chemical engineering, and its conformational transition dynamics was characterized as current fluctuations. The SiNW-FET devices were first measured to ensure the good FET performance. The single-molecule devices were measured by AFM/STORM to ensure the single-molecule modification. Three different devices were measured three times under each testing condition. The protein structure were adapted from RCSB PDB online (https://doi.org/10.2210/pdb1NKP/pdb).
Sampling strategy	An in-situ real-time electrical single-molecule detection has the capability of single-event tracking. The devices used in the experiments were measured three times under the same testing condition (temperature/concentration) to ensure the reproducibility of the data. Parallel experiments were performed three times on different devices to ensure the changing tendency.
Data collection	The current data were recorded by W.L., D.Y., Z.Y., and L.C. with the help of a HF2LI Lock-in Amplifier (Zurich Instruments) and a DL1211 preamplifier at a sampling rate of 57.6 or 28.8 kHz.
Timing and spatial scale	The data were collected from 2018.05.24 to 2022.04.04. The current signals were recorded after the modification of the device and collected within 24 hours. The devices were measured at Labs A725 and A727 in Beijing National Laboratory for Molecular Sciences, College of Chemistry and Molecular Engineering, Peking University, 292 Chengfu Road, Haidian District, Beijing 100871, P. R. China.
Data exclusions	No data are excluded from the analyses.
Reproducibility	Although values of the current levels varied from read to read among different SiNW-FET devices, the current patterns and the temperature dependence were highly reproducible. The current patterns are reproduced three times from at least three individual devices. The yield of the single-LC46 devices was ~10% on each chip.
Randomization	The I–t curves can be analyzed by the QuB software.
Blinding	We idealized the current fluctuations using the QuB software and measured the dwell times in each current level.

Did the study involve field work? ☐ Yes ☒ No

Reporting for specific materials, systems and methods

We require information from authors about some types of materials, experimental systems and methods used in many studies. Here, indicate whether each material, system or method listed is relevant to your study. If you are not sure if a list item applies to your research, read the appropriate section before selecting a response.

Materials & experimental systems

n/a	Involved in the study
☒	☐ Antibodies
☒	☐ Eukaryotic cell lines
☒	☐ Palaeontology and archaeology
☒	☐ Animals and other organisms
☒	☐ Clinical data
☒	☐ Dual use research of concern
☒	☐ Plants

Methods

n/a	Involved in the study
☒	☐ ChIP-seq
☒	☐ Flow cytometry
☒	☐ MRI-based neuroimaging
